# Mesh-based detailed skeletal models for the ICRP Reference Adults: Part 1. development and dosimetric impact

**DOI:** 10.1088/1361-6560/ae110f

**Published:** 2025-10-24

**Authors:** Chansoo Choi, Robert J Dawson, Yitian Wang, Bangho Shin, Johannes Tran-Gia, Maikol Salas Ramirez, Anna-Lena Theisen, Michael Lassmann, Wesley E Bolch

**Affiliations:** 1 J. Crayton Pruitt Family Department of Biomedical Engineering, University of Florida, Gainesville, FL, United States of America; 2 Department of Nuclear Medicine, University Hospital Würzburg, Würzburg, Germany; 3 Authors who contributed equally to this work and are co-first authors of the work.

**Keywords:** skeletal dosimetry, ICRP Reference Adults, mesh format, red bone marrow, endosteum

## Abstract

**Objective.:**

The skeleton is critical in radiation dosimetry. It is the tissue that houses both the red bone marrow (RBM) and the endosteum which are respectively linked to radiation-induced leukemia and bone cancer. The complex microstructure of these tissues provides challenges to dose assessment. Although detailed skeletal models have been developed, even the latest series of models remain voxel-based, thus limiting their anatomical and geometrical fidelity. The present study aims to develop the first mesh-based skeletal models aligned with the International Commission on Radiological Protection (ICRP) Reference Adult Male and Reference Adult Female to address these limitations.

**Approach.:**

A target skeletal dataset was first established through an extensive literature review to align with the ICRP Reference Adults while achieving anatomical realism. Primitive trabecular bone models were then generated from micro-computed tomography images using Fiji/ImageJ and Blender. These models were subsequently processed through an in-house automated C++/Python program, which adjusted their trabecular bone volumes, defined their endosteal layers, and partitioned the marrow into RBM and yellow bone marrow (YBM) to generate a final series of mesh-based skeletal models consistent with the target mass dataset.

**Main Results.:**

A total of 35 male and 38 female models of trabecular spongiosa were developed in a high-quality mesh format. Each model represents five distinct skeletal tissue regions: trabecular bone, and RBM and YBM within both the shallow (endosteal) and deep (non-endosteal) marrow. The models were designed to match the total skeletal tissue masses of the ICRP Reference Adults to within 0.5%. For selected cases, Monte Carlo simulations were performed by inputting them to the Particle and Heavy Ion Transport code System code together with the ICRP mesh-type reference phantoms, which showed that the improved model format enhanced specific absorbed fractions by up to 2.0-fold, while their anatomical refinement showed improvement by up to 1.6-fold.

**Significance.:**

The automated modeling techniques established here show strong potential for improvements in radiological protection as well as optimization of patient-specific marrow dosimetry in radiopharmaceutical therapy.

## 1. Introduction

The skeleton, viewed from a dosimetric perspective, is fundamentally composed of the cortical bone (CB), trabecular bone (TB), red bone marrow (RBM), and yellow bone marrow (YBM). Among these tissues, the RBM is critically important in dosimetry as it houses hematopoietic stem cells essential for blood cell production. It is associated with both a potential deterministic risk from patient treatments involving ionizing radiation and a long-term stochastic risk of radiogenic leukemia. Recognizing this significance, the International Commission on Radiological Protection (ICRP) assigned the RBM one of the highest tissue weighting factors of 0.12 among all radiosensitive tissues considered in effective dose calculations (ICRP 2007). Additionally, the endosteum, formerly known as the bone surface, is important in dosimetry and is also required for effective dose calculations, with an assigned tissue weighting factor of 0.01 (ICRP 2007). This ‘virtual’ marrow layer contains osteoprogenitor cells essential for bone growth, shaping, and maintenance, and has a potential risk for radiogenic bone cancer. It is assumed to be a thin layer located along the trabecular surfaces of the spongiosa as well as along the interior cortical surfaces of the medullary cavity of the long bones. Initially recommended to be 10 *μ*m in thickness (ICRP 1977, 1979) and widely adopted for several decades in the field of radiation dosimetry (Whitwell and Spiers 1976, Spiers 1988, Kramer et al 2006), the endosteum was later revised to a thickness of 50 *μ*m (Bolch et al 2007, ICRP 2009) based on new scientific evidence indicating that multipotent and totipotent mesenchymal precursors, located at a distance from the trabecular and cortical surfaces, can also serve as key target cells for bone cancer induction (Gössner et al 2000, Gössner 2003).

Nevertheless, the microscopic and complex structures of the skeleton have historically provided significant challenges for skeletal dosimetry. The field began in earnest in the 1960s through pioneering efforts by the research group at the University of Leeds, which utilized 2D chord-length-based skeletal models derived from optical scanning methods (Spiers 1968a, 1968b, 1969). Over the past couple of decades, substantial advancements have been made, with various research groups employing 3D image-based skeletal models, integrated with their own whole-body computational phantoms, that can be directly used for Monte Carlo simulations. The research group at the University of Florida (UF) has been a pioneer in image-based skeletal dosimetry since the early 2000s, developing skeletal models based on micro-computed tomography (*μ*CT) and nuclear magnetic resonance images. Among the most advanced models developed by UF are those based on *μ*CT images of spongiosa samples extracted from 32 skeletal sites of a 40 year male cadaver (Hough et al 2011) and 37 skeletal sites of a 45 year female cadaver (O’Reilly et al 2016). Similarly, research groups at the Federal University of Pernambuco and Tsinghua University acquired *μ*CT images from 8 skeletal sites in male and female cadavers (Kramer et al 2006, 2007) and 32 skeletal sites in a male cadaver (Gao et al 2017), respectively, for image-based skeletal dosimetry.

All previous image-based skeletal models, however, are in the voxel format, which presents several dosimetric limitations due to the fixed voxel resolution of *μ*CT images and their rigid, cuboidal structure. One major limitation is that the voxel resolution does not match the 50 *μ*m endosteal thickness, making precise definitions of the endosteum quite challenging [with resolutions of 30 *μ*m from UF (Hough et al 2011, O’Reilly et al 2016), 17.65, 30, and 60 *μ*m from the Federal University of Pernambuco (Kramer et al 2006, 2007), and 19 *μ*m from Tsinghua University (Gao et al 2017)]. Consequently, various approaches, such as adjusting the entire model’s resolution to 50 *μ*m (Hough et al 2011, O’Reilly et al 2016), have been implemented to approximate the endosteum. Another limitation stems from the cuboidal structure, which represents ideal curved boundaries between skeletal tissues as stepped, stair-like interfaces. This ‘voxel effect’ may lead to dose overestimation, particularly during irradiations with weakly penetrating radiation (Rajon et al 2002). Furthermore, the whole-body computational phantoms used in conjunction with these skeletal models are also in the voxel (or voxelized) format, leading to additional dosimetric limitations. For example, the limited voxel resolution prevents the cortical layer from fully enclosing regions containing the RBM and endosteum, leaving them partially exposed. This partial exposure can result in significant dose overestimation—up to thousands of times—again during irradiations with weakly penetrating radiation (Yeom et al 2016, Choi et al 2021).

Consequently, the present study aims to pioneer the development of detailed skeletal models in the mesh format, moving beyond the voxel format, by utilizing UF *μ*CT images to address the aforementioned dosimetric limitations. The mesh format is widely recognized as the most advanced and state-of-the-art modeling approach in computational dosimetry, surpassing the voxel format (Kainz et al 2019). The ICRP has also converted all reference computational phantoms from the voxel format (ICRP 2009, 2020a) to the mesh format (ICRP 2020b, 2024), addressing various dosimetric limitations, including incomplete CB enclosure (Yeom et al 2016, Choi et al 2021). To develop the skeletal models in conjunction with ICRP mesh-type reference computational phantoms (MRCPs), we first established a comprehensive target skeletal mass dataset aligned with ICRP Reference Adults, based on skeletal site-specific data gathered from diverse scientific sources. Primitive high-quality TB models in the mesh format were then created by converting and rendering the *μ*CT images using Fiji/ImageJ (Schindelin et al 2012) and Blender 4.2 software. Next, a fully automated modeling pipeline was developed using in-house C++ and Python programs, built upon the Computational Geometry Algorithms Library (CGAL, www.cgal.org/). This pipeline was employed to adjust the TB volume (TBV), generate a true and continuous 50 *μ*m-thick endosteal layer, and systematically partition the marrow regions into RBM and YBM regions, in accordance with the target skeletal data. Finally, to assess the dosimetric impact of the new skeletal models, absorbed fractions (AFs) and specific AFs (SAFs) were calculated for selected skeletal sites and analyzed in relation to improvements in model format (mesh vs. voxel) and anatomical conformity to the target skeletal data (fitted vs. unfitted).

## 2. Materials and methods

### 2.1. *μ*CT images from the UF

Table 1 provides detailed information on the comprehensive set of UF *μ*CT images utilized in the present study to develop detailed skeletal models in the mesh format. For the adult male, the images were obtained from spongiosa samples at 32 skeletal sites in a 40 year-old male cadaver (170 cm in height and 82 kg in body weight) who died due to sudden complications of cardiopulmonary arrest following a myocardial infarction (Hough et al. 2011). For the female, the images were collected from 37 skeletal sites in a 45 year-old female cadaver (additional details unknown) (O’Reilly et al 2016). All images were acquired using a *μ*CT-80 system from Scanco Medical AG (Bassersdorf, Switzerland) with an isotropic voxel resolution of 30 *μ*m. Due to the size constraints of *μ*CT imaging, these samples do not capture entire skeletal sites; however, their fine, repetitive structural patterns are considered representative, as accepted in previous image-based skeletal modeling studies. From these *μ*CT images, two distinct regions—the TB and marrow (containing both the RBM and YBM) regions—were segmented. The segmentation process involved enhancing the signal-to-noise ratio, employing a median filter, and setting a determined threshold value for the gray level, resulting in binary images segmented into the TB and marrow regions. This entire segmentation process was conducted using an ITK-based program (Rajon et al 2006). In the previous studies (Hough et al 2011, O’Reilly et al 2016), the resolution of these images was decreased to 50 *μ*m to match the endosteal thickness for the development of skeletal models in the voxel format. In the present study, however, the original *μ*CT resolution of 30 *μ*m was retained to preserve a faithful representation of trabecular anatomical structures.

### 2.2. MRCPs of ICRP Publication 145

**Figure 1.**
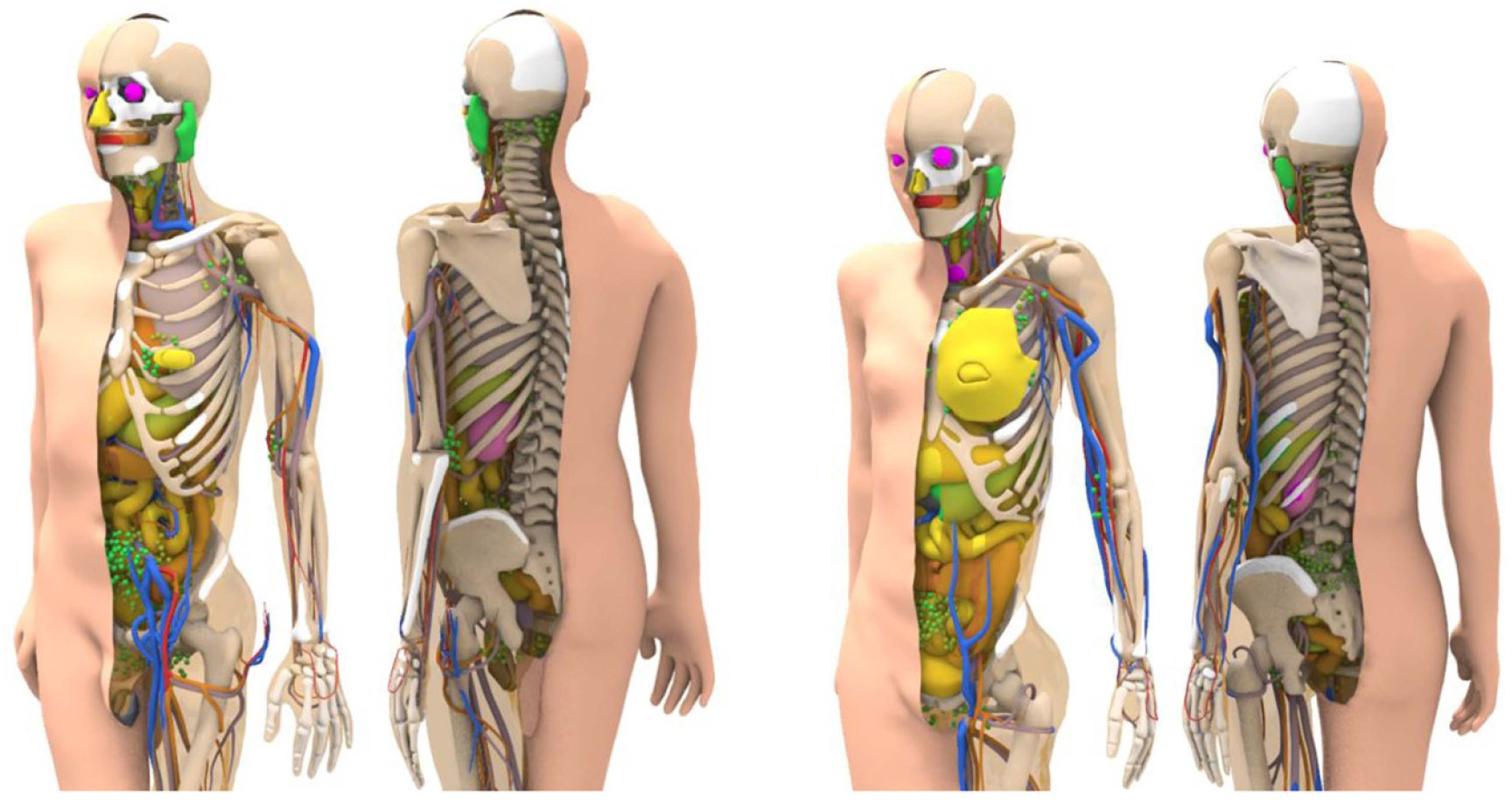
Mesh-type reference computational phantoms (MRCPs) of ICRP Publication 145 (ICRP 2020b): male (left) and female (right).

Figure 1 shows the MRCPs of ICRP Publication 145 (ICRP 2020b), the whole-body computational phantoms with which the detailed skeletal models developed in the present study will eventually be combined for skeletal dosimetry. The body dimensions (height and body weight) and organs/tissues of the MRCPs are aligned with the data for the ICRP Reference Adults provided in ICRP Publication 89 (ICRP 2002), with all organs/tissues implicitly assumed to uniformly contain their blood content. Most organs/-tissues were directly converted from the voxel-type reference computational phantoms (VRCPs) of ICRP Publication 110 (ICRP 2009), while certain small, thin, or complex structures, such as the cortical layer enclosing the interior skeletal tissues, were further enhanced. Additionally, the MRCPs include micron-thick radiosensitive target regions within the skin, eye lens, urinary bladder, alimentary tract organs, and respiratory tract organs, as prescribed by the ICRP (ICRP 1994, 2006, 2010). The MRCPs explicitly define all organs/tissues required for effective dose calculations (ICRP 2007), with only two exceptions—RBM and endosteum—which are implicitly represented within the spongiosa and/or medullary cavity. The male MRCP consists of 2.5 million triangular facets in the polygonal mesh (PM) format and 8.2 million tetrahedra in the tetrahedral mesh (TM) format, while the female MRCP contains 2.6 million triangular facets in PM format and 8.2 million tetrahedra in TM format. Initially created in PM format, which can be directly imported into 3D modeling software and algorithms, the MRCPs were eventually converted to TM format, which can be directly implemented in Monte Carlo codes (ICRP 2020b) (this PM-to-TM workflow was similarly applied in the present study).

### 2.3. Establishment of target skeletal data

#### 2.3.1. Inconsistency in skeletal data within the ICRP Reference Adults

The detailed skeletal models developed in the present study aim to enhance the representation of skeletal tissues within the spongiosa of the MRCPs (ICRP 2020b) and ultimately to serve as representative models for ICRP Reference Adults in conjunction with the MRCPs. Thus, consistency between the skeletal data in these models and the MRCPs is essential. However, due to differing derivation processes, the skeletal data from the UF *μ*CT images and the MRCPs are not presently in alignment. The *μ*CT images were obtained from spongiosa samples across various skeletal sites in individual male and female cadavers, segmented into the TB and marrow (containing both the RBM and YBM) regions, reflecting each unique anatomical characteristics and potential individual biases. On the other hand, the spongiosa of the MRCPs was developed through a different methodology, nearly identical to that used for the VRCPs (ICRP 2009, Yeom et al 2016). This process involved: (1) segmenting spongiosa from whole-body CT images, (2) volumetric scaling to align the total skeletal mass with ICRP data from ICRP Publication 89 (ICRP 2002), and (3) deriving skeletal masses at specific skeletal sites based on factors such as the RBM distribution and the marrow cellularity factor (CF) of ICRP Publication 70 (ICRP 1995). Note that the CF represents the proportion of haematopoietically active volume, that is, the volume of RBM, within the total marrow volume of each skeletal site. Consequently, directly integrating the *μ*CT images into the MRCPs inevitably leads to discrepancies with the ICRP data (see table 2), which is highly significant and widely applied throughout the field of radiation protection, including within the ICRP’s broad scope of work.

#### 2.3.2. Collection of skeletal data through literature review

Prior to developing the skeletal models based on the UF *μ*CT images, we established target skeletal data for each site, grounded in anatomically reasonable evidence, to ensure consistency with the ICRP data (ICRP 2002) when these models are integrated into the MRCPs. To achieve this, we conducted an extensive literature review focusing on the bone volume fraction (BV/TV), a widely reported parameter in the literature that represents the ratio of TBV to total spongiosa volume at each skeletal site. Although derived from individual male and female cadavers, the UF *μ*CT images were acquired using one of the most advanced techniques and provide their own BV/TV values. To further refine these BV/TV values, we conducted a systematic electronic search across multiple databases, including PubMed, Web of Science, and Google Scholar, to identify relevant studies that provide BV/TV values for each skeletal site, based on strict criteria: (1) acquisition through *μ*CT scanning, (2) derivation from multiple cadavers, and (3) values unadjusted by compression testing or similar modifications. Table 3 summarizes these studies, detailing the skeletal sites where BV/TV values are available. Note that while most studies provide pooled BV/TV values regardless of sex, in cases where sex-specific values were available, the present study incorporated them accordingly.

As shown in table 3, some of the gathered studies provide BV/TV values for similar skeletal sites, with certain studies offering values at the same level of detail and others at varying levels of image resolution. In such cases, BV/TV values used in the present study were selected according to specific criteria. For skeletal sites with the same level of detail, preference was given to the study involving a larger number of cadavers to reduce the potential bias introduced by inter-individual anatomical variability in skeletal structures. For example, both Parsa et al (2015) and Van Dessel et al (2013) provide BV/TV values for the mandible, but values from Parsa et al (2015) were selected due to their larger sample size. Where levels of detail varied, BV/TV values for the most specific skeletal site within practical applicability were prioritized. For instance, while some studies (Bevill et al 2006, Hulme et al 2007) provide BV/TV values for entire vertebrae, others (Schröder et al 2021, 2022) specify lumbar vertebrae, with a few (Ulrich et al 1999, Eckstein et al 2007, Perilli et al 2012) focusing on the 2nd lumbar vertebra. Since the 2nd lumbar vertebra represents a practical level of detail, BV/TV values specific to this site were selected. Among these, values from Eckstein et al (2007), which used the largest number of cadavers for the measurement, were ultimately chosen. As a contrasting example, while Moon et al (2004) offer BV/TV values for highly detailed substructures of the mandible, values for the entire mandible (Parsa et al 2015) were selected instead due to practical limitations. Although studies for some skeletal sites were unavailable, the compiled BV/TV values served as reference points for establishing skeletal data based on anatomically reasonable evidence.

#### 2.3.3. Establishment of target skeletal data aligned with ICRP Reference Adults

To align with ICRP reference data (ICRP 2002), the primitive BV/TV values derived from the UF *μ*CT images were adjusted. As shown in table 2, this adjustment requires an overall increase of 4.4% for the male and an overall decrease of 27.5% for the female. To minimize distortions at specific skeletal sites, a uniform adjustment was initially applied across all sites. The adjusted BV/TV values were then compared to the compiled BV/TV data (see section 2.3.2) to enhance anatomical accuracy. For skeletal sites where the adjusted values fell outside one standard deviation from the mean of the compiled data, further refinement was applied to bring them within this range. As this process inevitably reintroduced minor inconsistencies with the ICRP data, the total TB mass was re-estimated by applying the refined values to the spongiosa of the MRCPs. Subsequently, the uniform adjustment and comparison/refinement steps were iterated until full alignment with the ICRP data was achieved. The determined target BV/TV values, while not fully reproducing the actual anatomy for skeletal sites lacking compiled data, maintain anatomical plausibility in these sites by applying minimal distortion to the UF *μ*CT-derived values, while ensuring high anatomical accuracy for sites with compiled data.

As the target BV/TV values were determined for all skeletal sites, the remaining region, i.e. the marrow region, was automatically defined. To further subdivide this marrow region into RBM and YBM, CF values from ICRP Publication 70 (ICRP 1995) were applied, with a uniform adjustment made to ensure consistency with the ICRP data (ICRP 2002). This adjustment resulted in an overall increase of 10.9% for the male and 8.8% for the female. Table 4 presents the target BV/TV values and CF values for each skeletal site, as configured to achieve full alignment with the ICRP data when integrated into the MRCPs. We note that for some skeletal sites, the availability of more precise data allowed the original *μ*CT images, which represented 32 and 37 skeletal sites for the male and female, respectively, to support the development of 35 and 38 detailed mesh-based skeletal models.

### 2.4. Development of mesh-based detailed skeletal models

#### 2.4.1. Conversion of μCT images to primitive TB models

**Figure 2.**
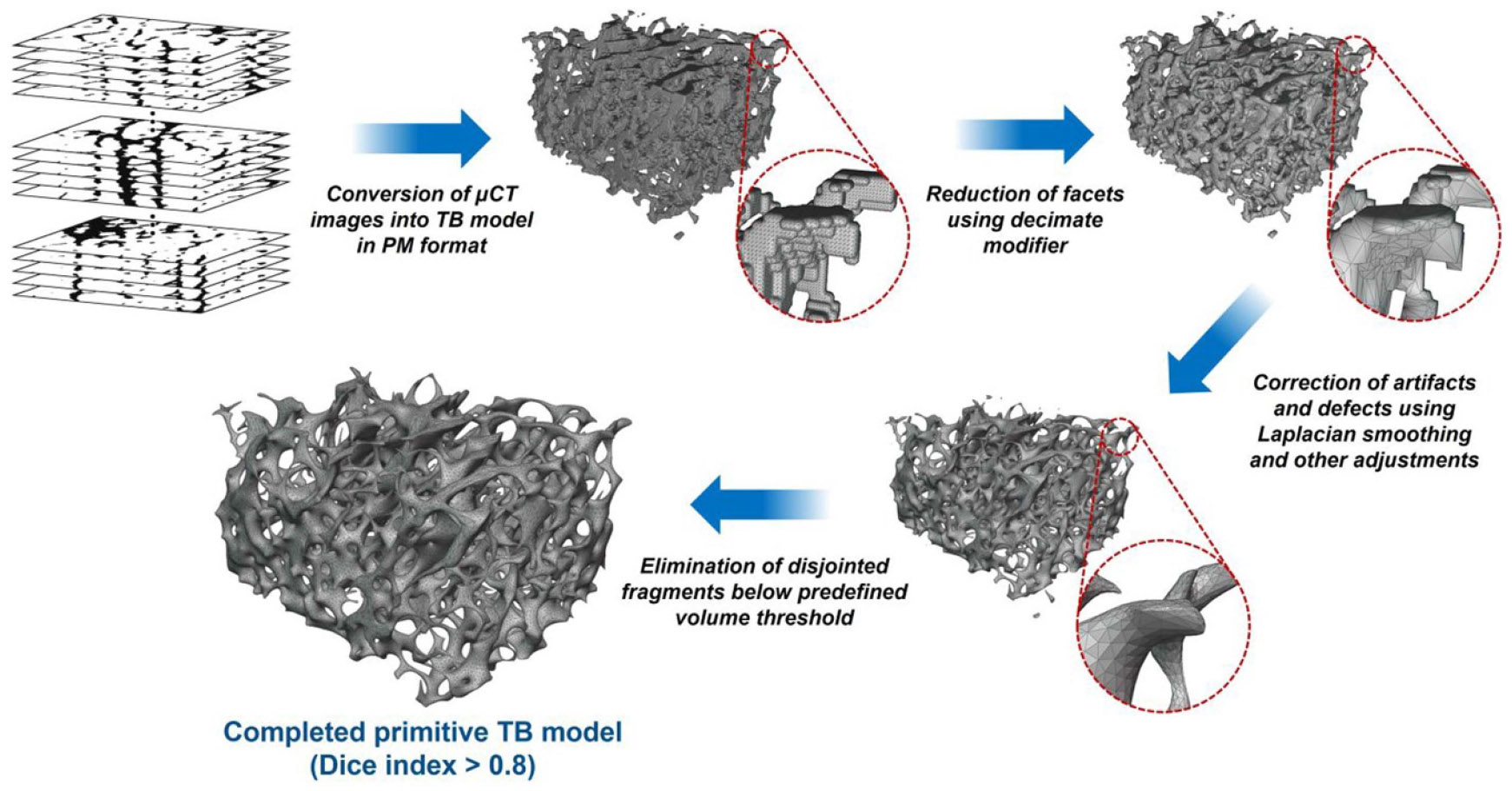
Procedure for generating primitive trabecular bone (TB) models in polygonal mesh (PM) format. The figure uses the female sacrum as an example.

As the first step in developing detailed skeletal models, primitive TB models in PM format were generated from the UF *μ*CT images to serve as foundational inputs for subsequent steps outlined in section 2.4.2. Figure 2 schematically demonstrates the generation process. The *μ*CT images, already segmented (see section 2.1), were imported into the Fiji distribution of the open-source ImageJ software (Schindelin et al 2012) and directly exported as TB models in PM format. These models were then imported into Blender 4.2, an open-source 3D modeling software, for processing to enhance their structural quality and fidelity. This process began with the application of the Decimate Modifier feature to reduce the number of facets, enhancing computational efficiency, followed by the Global Laplacian Smoothing feature to remove stair-like surface artifacts while preserving the original volume as much as possible. Next, the 3D Print Toolbox Extension feature was employed to evaluate volume changes relative to the original and to identify PM defects such as intersecting faces and non-manifold edges. Then, several adjustments, including the use of the Local Laplacian Smoothing feature, were applied to restore the original volume and resolve detected defects. To address potential imaging artifacts, present in the *μ*CT images, a Blender Python API script was written and used to identify and remove disjointed fragments smaller than a predefined volume threshold of 0.000108 mm^3^ (equivalent to the total volume of four single voxels). Throughout this process, the Dice index (Dice 1945)—a metric measuring the overlapping fraction between two objects—was monitored using an in-house C++ program to validate the accuracy of the conversion.

All models were refined again in Blender, including several adjustments such as the Local Laplacian Smoothing feature, to achieve a Dice index above 0.8, ensuring that the completed primitive TB models accurately reflected the structure and volume of the *μ*CT images while being converted into the high-quality PM format.

#### 2.4.2. Automated development of skeletal models from primitive models

**Figure 3.**
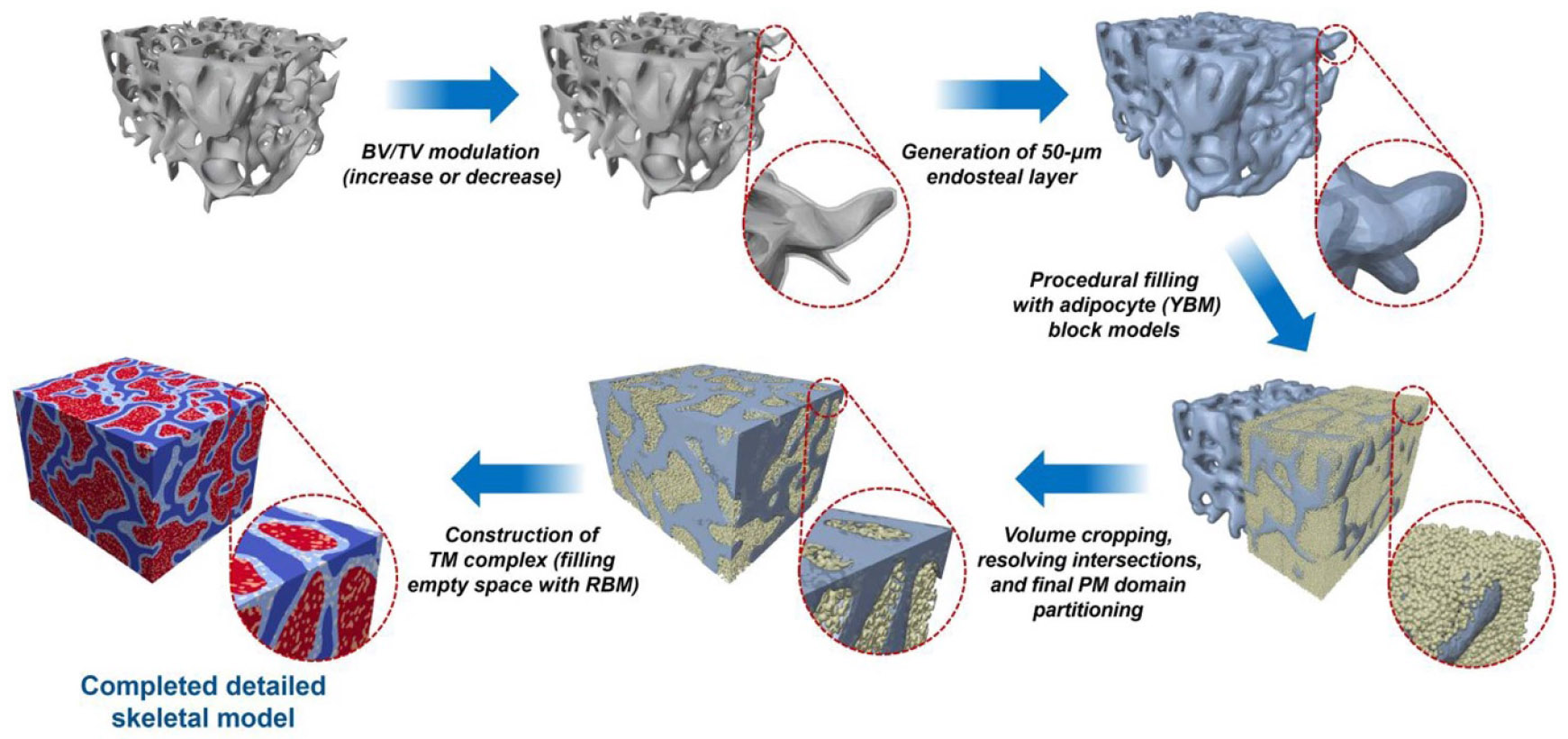
Schematic overview of the automated workflow for transforming primitive TB models in PM format into finalized detailed skeletal models in TM format. The figure uses the male C3 as an example.

Following the generation of primitive, smooth TB models in PM format, they were entered into a sequential, multistep workflow to refine and incorporate not only the TB, but also the endosteum, RBM, and YBM, in alignment with the target skeletal data, ultimately completing the detailed skeletal models in TM format. This modeling workflow was fully automated using in-house C++ and Python programs, with a detailed description provided in the **Electronic Annex** to this study. Figure 3 presents a schematic overview of this workflow.

Each of the primitive TB models in PM format underwent the following modeling process. First, the surface of the TB model was refined to achieve the target BV/TV value specific to each skeletal site. When an increase in BV/TV was required, the 3D Alpha Wrapping algorithm in CGAL was executed multiple times in parallel with varying offset values to generate several outcomes, and the optimal result, determined based on BV/TV calculations, was carried forward to subsequent steps. Conversely, when a reduction in BV/TV was necessary, a more complex procedure was required, as the 3D Alpha Wrapping algorithm, due to its inherent nature, cannot be directly used for bone trabeculae volume reduction. For this, points were uniformly generated within the TB model through point sampling routine (Rocchini and Cignoni 2000), and the minimum (normal) point-to-surface distance between each query point and the TB surface (TBS) was computed. This process was repeated multiple times, each with a different prescribed rejection threshold distance, which was compared to the minimum distance for each query point, and thus points falling within the threshold distance were rejected at each iteration. In this way, ‘layers’ of varying thicknesses were removed from the outer boundary of the TB point cloud. The point clouds generated in each iteration were then used to create new TBSs through the 3D Alpha Wrapping algorithm, from which the final surface was selected based on BV/TV calculations. With the refined TB model, an endosteal layer was created by applying the 3D Alpha Wrapping algorithm with a fixed 50 *μ*m offset. Additional ‘shell’ layers for the TB and endosteal surfaces, necessary to prevent boundary ambiguity later in the workflow, were generated in a similar manner with a 1 *μ*m offset.

To replicate the native structure of YBM, in which spherical adipocytes (fat cells) are clustered together, the marrow region of the model inherited from the previous step was filled with numerous adipocyte models. To avoid the computational inefficiency of generating these adipocyte models ‘on the fly’ for each skeletal site, a large library of pre-generated adipocyte block models was established procedurally. The size distribution of individual adipocyte models within each block model followed an empirical Gaussian distribution model described by Rozman et al (1990), which is parameterized by the fat fraction (assumed to equate to 1—CF) specific to each skeletal site. Each block model was created by filling a 2 × 2 × 2 mm^3^ region with numerous icosahedral adipocyte models, whose radii were sampled from the Gaussian distribution. The positions of these adipocyte models were semi-randomly determined using a 3D implementation of Poisson disk sampling based on the Bridson algorithm (Bridson 2007, Gamito and Maddock 2009, Ebeida et al 2011), ensuring minimal overlap among individual adipocyte models. Each generated block model was uniquely labeled according to its associated fat fraction for reference during the modeling workflow.

For each model, following TB thickness adjustment and endosteum definition, the top three adipocyte block candidates were selected based on their closeness to the user-prescribed target fat fraction. Each selected block was sequentially placed and Boolean-united with the others, progressively expanding the adipocyte structure until the entire bounding box of the model was fully covered. The extended structure was then further refined by performing additional Boolean operations with the previously generated ‘shell’ layers to remove adipocyte regions overlapping the TBV and to partition the remaining adipocyte regions into shallow and deep YBM, located inside and outside the endosteal layer, respectively. Any remaining unassigned space within shallow and deep marrow, where the YBM was not defined, was designated as RBM. The final PM model, now divided into five distinct regions of interest, was subsequently tetrahedralized using CGAL’s 3D polyhedral mesh complex class and exported as a VTU file for visualization in ParaView (www.paraview.org/). The mesh vertex and connectivity information contained in the VTU file was then converted into the NODE/ELE format, enabling compatibility with Monte Carlo radiation transport codes such as the Particle and Heavy Ion Transport code System (PHITS).

### 2.5. Monte Carlo simulations

To evaluate the dosimetric impact of the detailed mesh-based skeletal models, the male proximal humeri and female T6 were selected as representative skeletal sites, and their electron AFs and SAFs were computed using Monte Carlo radiation transport simulations with PHITS version 3.24 (Sato et al 2018, 2024). Note that the AF is the fraction of emitted energy absorbed in the target region, and the SAF is the AF normalized by the mass of the target region. The calculated values were then compared with those obtained from voxel models and unfitted mesh models to assess the effects of geometrical and anatomical refinements, respectively. For this, the voxel models were produced by voxelizing the mesh models at a 50 *μ*m resolution using an in-house C++ program, while the unfitted mesh models were generated using the same process outlined in section 2.4.2 with the original cadaver-specific BV/TV and ICRP reference CF values preserved. Note that a 50 *μ*m resolution was chosen for the voxel models to preserve the 50 *μ*m-thick endosteal layer and to focus solely on the effects of the model format. As in the previous study (O’Reilly et al 2016), the AFs were calculated using the following equation, which combines the micro- and macrostructural AFs:

(1) $$\phi \;(r_{T}\leftarrow r_{S},E_{i})=\phi_{\mu }\left(r_{T}\leftarrow r_{S},E_{i}\right)\phi_{M}\left(\text{spongiosa}\leftarrow r_{S},E_{i}\right)$$

where *ϕ_μ_* (*r*_T_ ← *r*_S_, *E*_i_) is the AFs for the target (*r*_T_) and source (*r*_s_) regions obtained from microstructural transport runs (infinite spongiosa) using the detailed skeletal models for a given energy *E*_i_, capturing the detailed representation of skeletal tissues, while *ϕ*_M_ (spongiosa ← *r*_S_, *E*_i_) is the corresponding AFs derived from macrostructural transport runs using the MRCPs of ICRP Publication 145 (ICRP 2020b) for the same energy, accounting for electron escape beyond the spongiosa. A more detailed discussion of this combination method can be found in O’Reilly et al (2016). Then, the SAFs were obtained by dividing the final AFs by the mass of the corresponding target region. Note that the densities and elemental compositions of TB, RBM, and YBM were based on values provided in the International Commission on Radiation Units and Measurements (ICRU) Report 46 (ICRU 1992), with modifications to reflect the assigned blood contents and miscellaneous tissues within each skeletal tissue. The PHITS code configurations used for the AF and SAF calculations in the present study are listed in table 5. All simulations were performed on the UF’s high-performance computing cluster, HiPerGator 3.0 (www.rc.ufl.edu/about/hipergator/).

## 3. Results

### 3.1. Mesh-based detailed skeletal models

**Figure 4.**
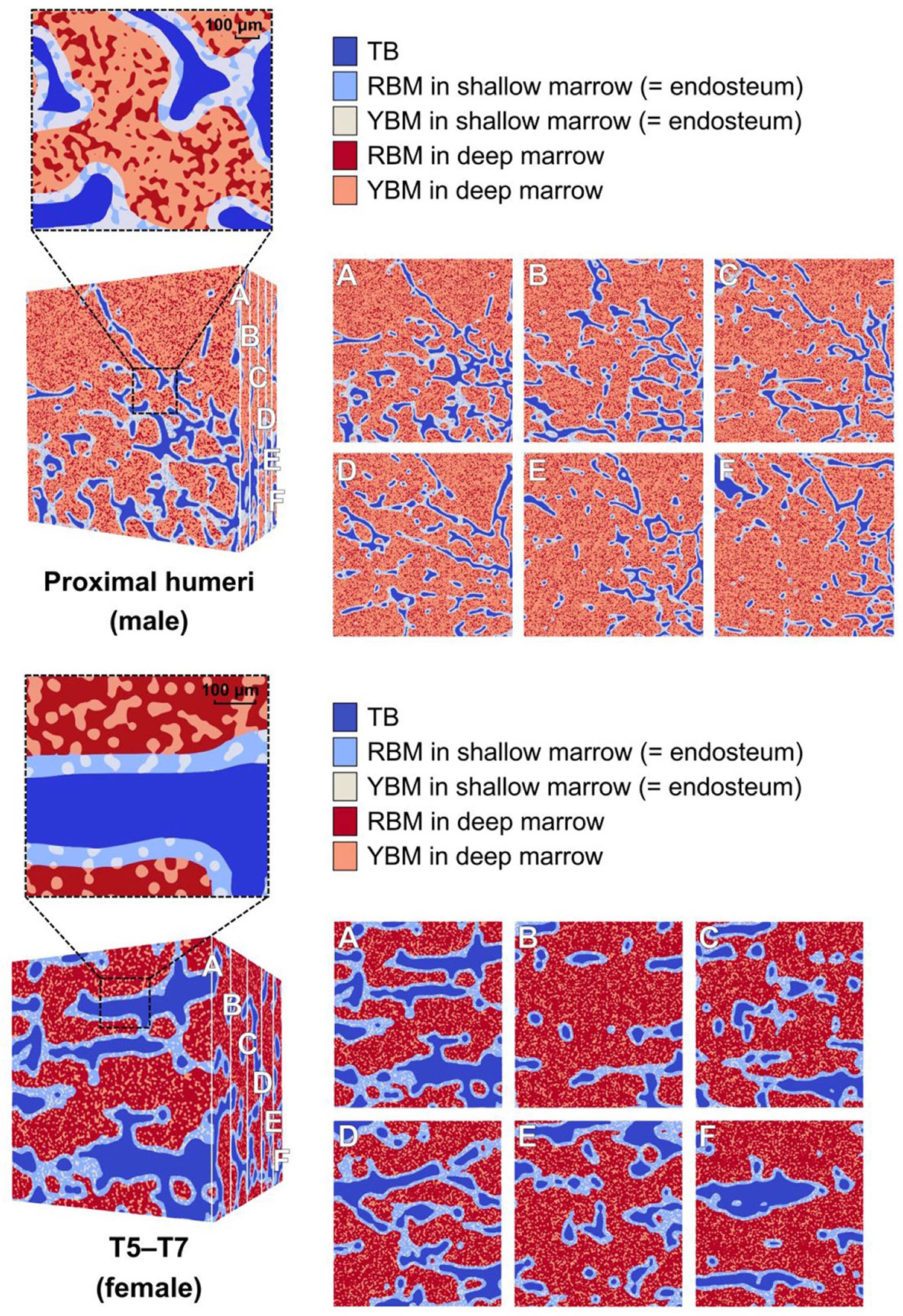
Representative examples of mesh-based detailed skeletal models developed in this study: male proximal humeri model (top) and female T5–T7 model (bottom). The bone volume fraction (BV/TV) and cellularity factor (CF) of the developed male proximal humeri model are 0.087 and 0.271, respectively (reference values: 0.088 and 0.277). For the developed female T5–T7 model, the BV/TV and CF are 0.160 and 0.757, respectively (reference values: 0.160 and 0.761).

**Figure 5.**
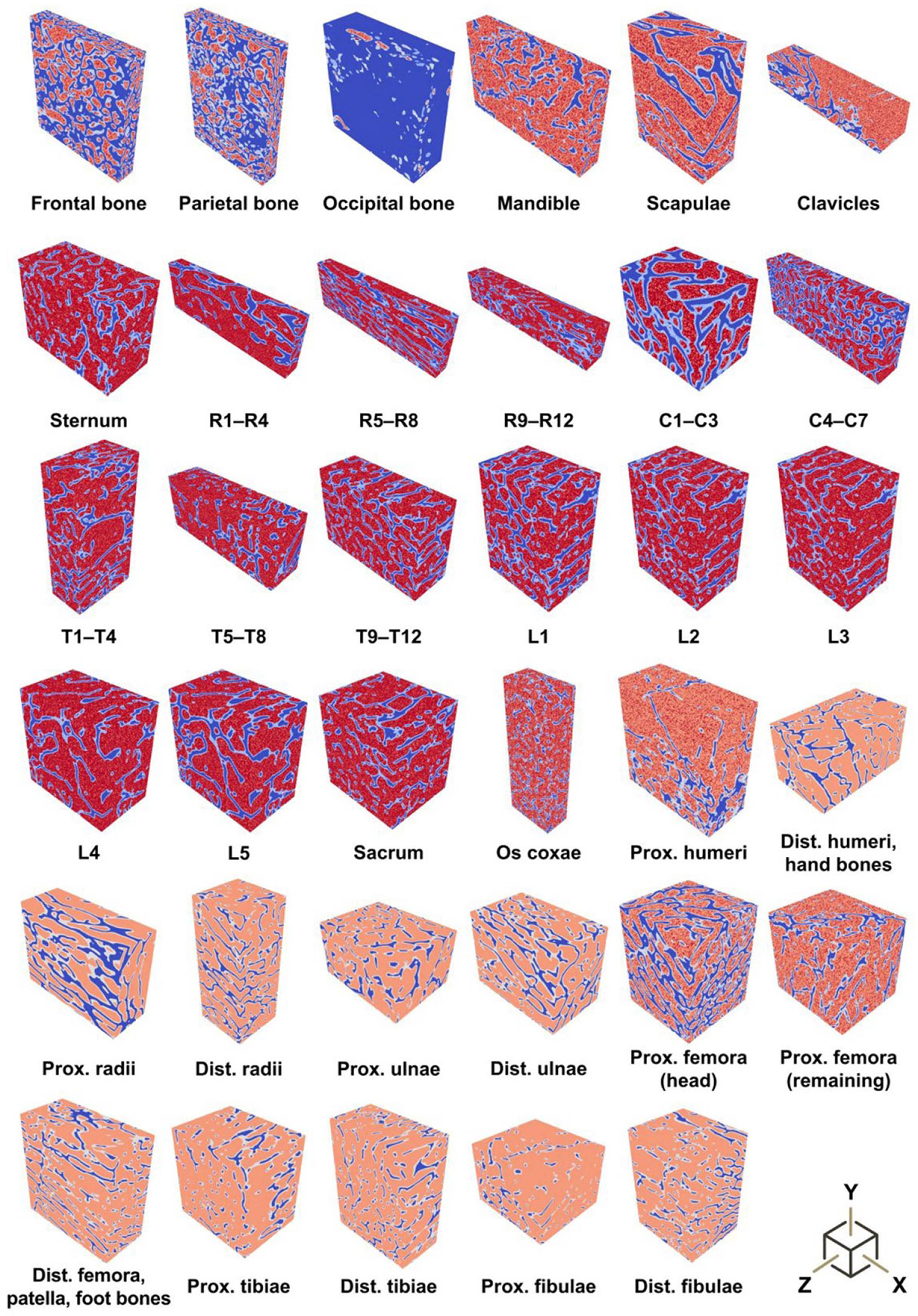
Overview of the complete set of mesh-based detailed skeletal models for the male.

**Figure 6.**
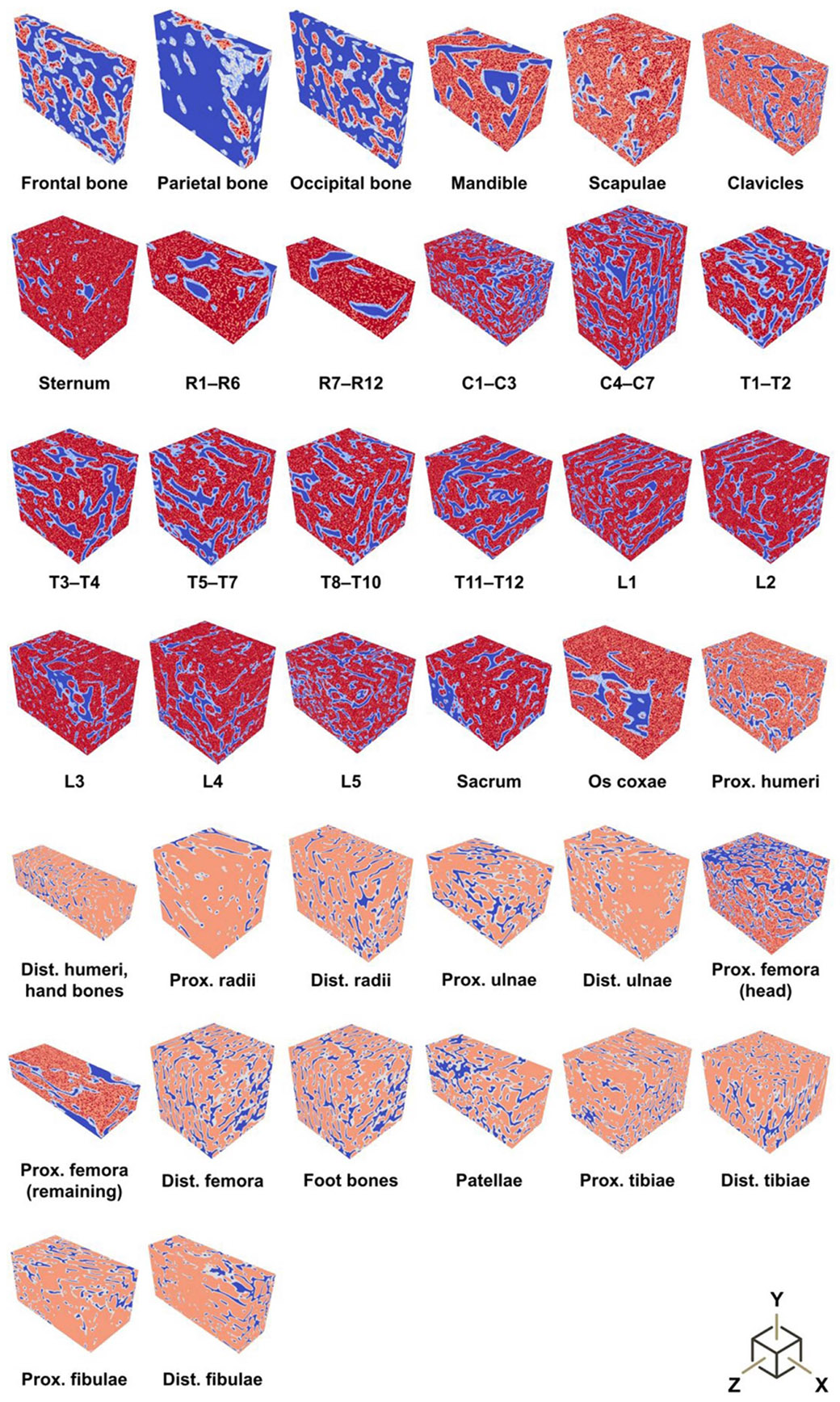
Overview of the complete set of mesh-based detailed skeletal models for the female.

In the present study, we developed 35 and 38 detailed mesh-based skeletal models for the ICRP reference adult male and female, respectively, using the *μ*CT images from 32 and 37 skeletal sites. The increase in the number of skeletal models was due to certain *μ*CT images being expanded into multiple models in accordance with the more subdivided target skeletal dataset (see table 4). Figure 4 presents detailed views of the skeletal models, exemplified by the male proximal humeri model and the female T5–T7 model. As shown in the figure, the skeletal models are divided into five distinct regions: TB, RBM and YBM within the shallow marrow (inside the endosteum), and RBM and YBM within the deep marrow (outside the endosteum). A 50 *μ*m endosteal layer is precisely defined in these models. Figures 5 and 6 present an overview of the complete set of skeletal models for the male and female, respectively.

All developed skeletal models show good agreement with the target BV/TV and CF values listed in table 4, exhibiting an average of 1.0% relative difference (maximum 3.9%) and 1.0% (maximum 3.7%) for the male, and 1.1% (maximum 3.2%) and 0.7% (maximum 3.3%) for the female. Note that skeletal sites with the target CF values of zero were excluded from the calculation of CF differences. When integrated into the MRCPs (ICRP 2020b), compensatory effects—where slightly higher and lower values across skeletal sites offset each other—further reduced these differences, resulting in excellent agreement with the target TB, RBM, and YBM masses from ICRP data (ICRP 2002) as shown in table 6. This marks a clear improvement over the differences seen in table 2, where the *μ*CT images were directly integrated without adjustment to both trabeculae thickness or the marrow fat fraction. This high level of agreement with the target values minimizes potential biases in skeletal site representativeness that could stem from the limited sample size of the *μ*CT images, thereby further reinforcing the representativeness of the developed models.

**Figure 7.**
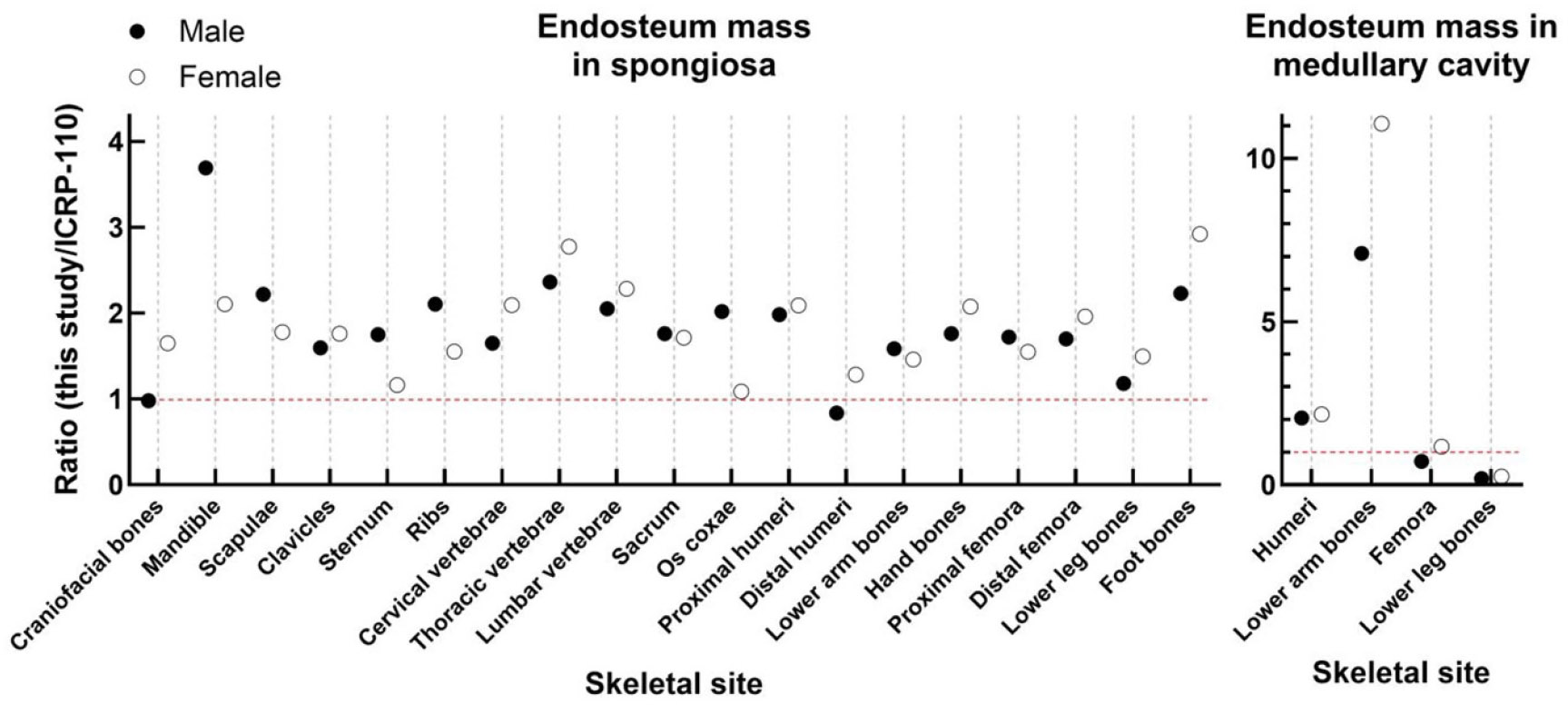
Ratios of the endosteal masses measured in the present study to those provided in ICRP Publication 110 (ICRP 2009) for each skeletal site.

Table 7 provides the masses of the target tissues, RBM and endosteum, measured for each skeletal site in the present study. To provide a comprehensive set of their masses, the endosteum within the medullary cavity, which was not the focus of this study, was also included by simply defining 50 *μ*m-thick endosteal layers along the interior cortical surfaces of the medullary cavity and measuring their masses. Figure 7 compares the endosteal masses measured in this study with those provided in ICRP Publication 110 (ICRP 2009). Note that the ICRP-110 values were derived from the measurements reported by Bolch et al (2007), which were obtained from *μ*CT images of a 66 year-old male cadaver, and were applied to both sexes; these values have remained unchanged since their initial publication and continue to serve as the basis for all subsequent ICRP calculations (ICRP 2010, 2016). As shown in figure 7, the updated endosteum masses obtained in the present study were substantially larger than the ICRP-110 values for most skeletal sites, resulting in much greater total masses (this study: male 891.4 g, female 704.9 g; ICRP Publication 110: male 544.4 g, female 407.5 g). Unlike the previous measurements, the present study considered male and female separately, incorporated adjustments based on ICRP reference data and multiple anatomical considerations and derived the values from endosteum precisely defined in an improved mesh format. These methodological refinements are expected to ensure consistency with ICRP calculations while enhancing anatomical representativeness, thereby providing an updated dataset that can serve to complement the existing values.

To represent their microscopic complexity and detailed internal structures, each of the developed skeletal models consists of a substantially large number of tetrahedra—significantly more than those used in previously developed dosimetry models in TM format (e.g. ~8.2 million for the male MRCP and ~8.6 million for the female MRCP). Each male skeletal model contains an average of ~160 million tetrahedra for skeletal sites with nonzero CF values and ~4.5 million for those with zero CF values. Each female model contains an average of ~86 million tetrahedra for sites with nonzero CF values and ~5.2 million for those with zero CF values. The lower tetrahedral counts observed in sites with zero CF values are due to the marrow region being entirely filled with the YBM, eliminating the need to model numerous individual adipocytes, which would otherwise require many tetrahedra.

### 3.2. Dosimetric impact

To investigate the dosimetric impact of the developed mesh-based skeletal models from two distinct perspectives, i.e. model format (mesh vs. voxel) and alignment with the target skeletal data (aligned vs. nonaligned), the AFs and SAFs were calculated for the male proximal humeri and female T6 as representative cases. Source regions include the TBS, TBV, RBM, and YBM, while target regions are the RBM and endosteum. All the values were calculated with statistical relative errors within 1%.

#### 3.2.1. Impact of model format

**Figure 8.**
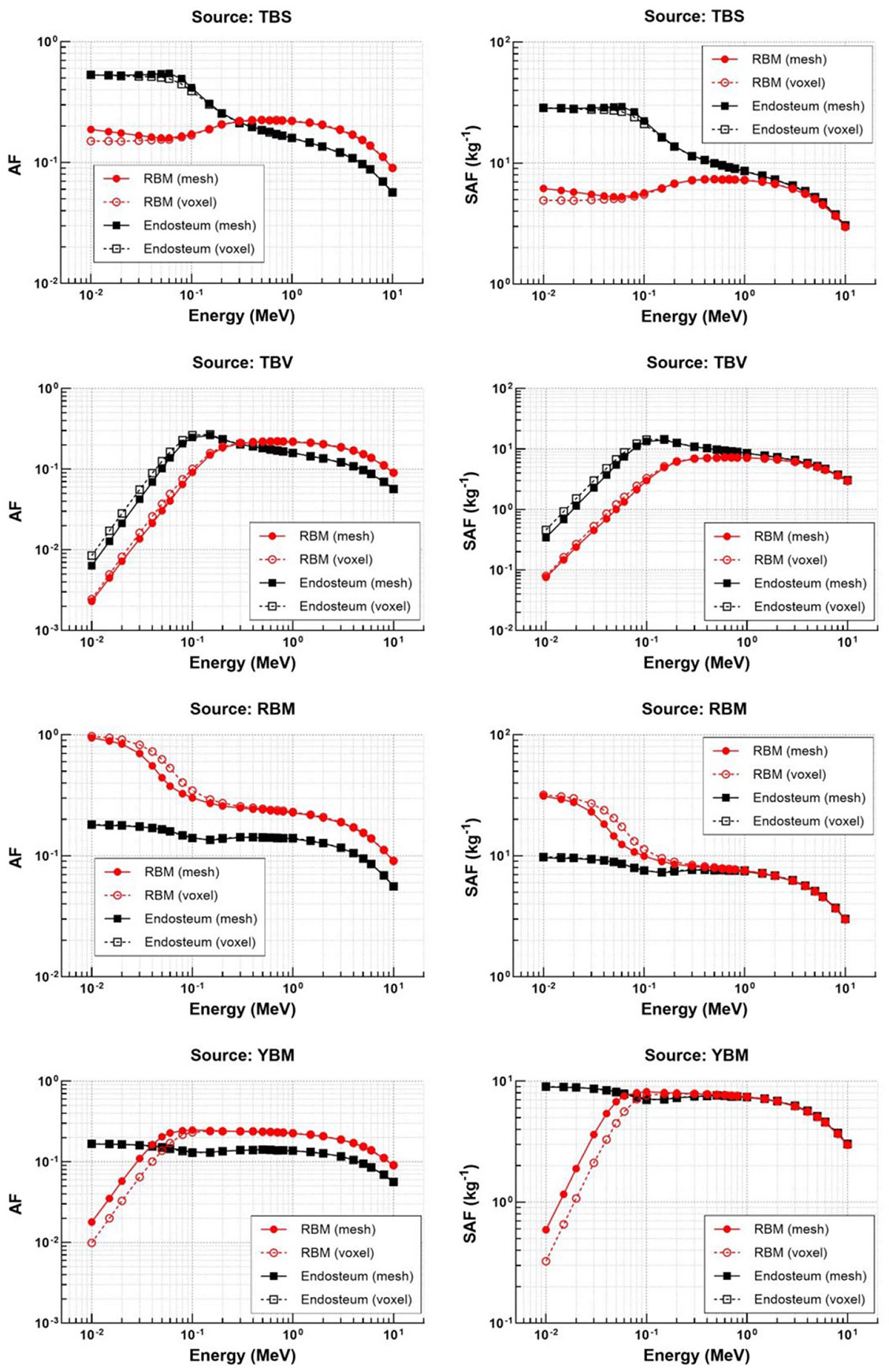
Absorbed fractions (AFs) and specific absorbed fractions (SAFs) calculated using the mesh-based detailed skeletal model (‘mesh’) and its voxelized model (‘voxel’) of the male proximal humeri. AFs are shown on the left, SAFs on the right. Source regions are the trabecular bone surface (TBS), trabecular bone volume (TBV), red bone marrow (RBM), and yellow bone marrow (YBM), while target regions are the RBM and endosteum.

Figure 8 compares the values calculated for the male proximal humeri using the mesh model and its voxelized counterpart with a 50 *μ*m resolution. As the voxel model preserves the masses of all regions from the mesh model within 1%, the trends observed in the SAF differences closely mirror those of the AFs. When the TBS and endosteum are the source and target regions, respectively, the two models show good agreement across the entire energy range, with maximum differences remaining within 10%. For this combination, the low-energy electron AFs have traditionally been approximated as 0.5, assuming equal energy deposition in both the TB and endosteum by electrons originating at the TBS (ICRP 2023). The present study yields similar values (~0.53 at 10 keV) from both mesh and voxel models, with the slight difference attributed to the anatomically irregular and curved geometry of the TBS.

**Figure 9.**
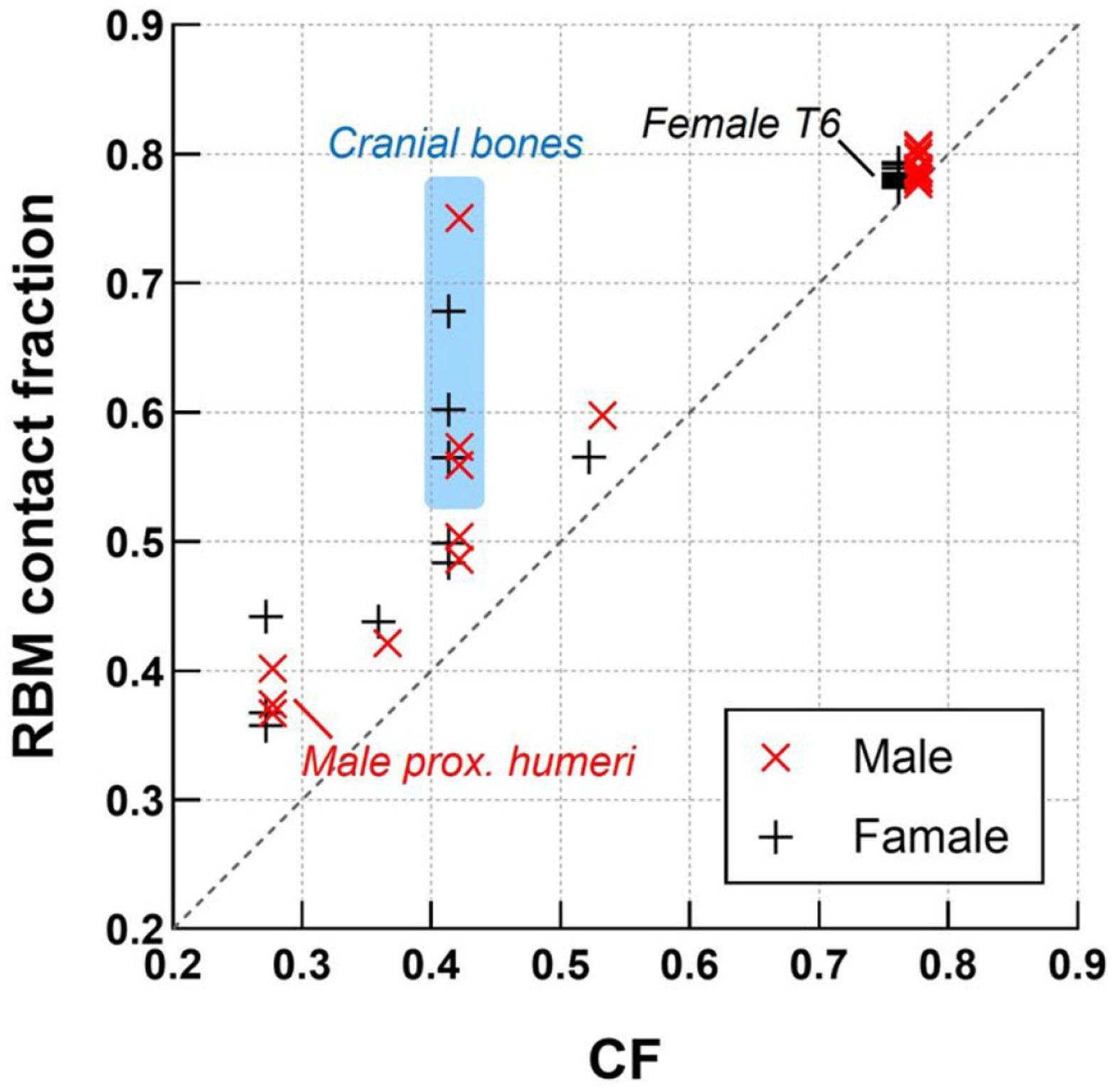
Contact fractions of red bone marrow (RBM), defined as the fraction of the trabecular bone (TB)–marrow interface occupied by RBM, in the developed mesh-based detailed skeletal models (male: red ×; female: black +). In all cases, the RBM contact fractions exceed the corresponding cellularity factors (CFs), with the difference becoming more pronounced at lower CF values—except in the cranial bones. The cranial bones exhibit significantly higher bone volume fraction (BV/TV) compared to other skeletal sites, resulting in notably elevated RBM contact fractions. Skeletal sites with a CF of zero were excluded from this figure.

For cases with the RBM as the target region, the voxel model underestimates the values by up to 20% at low energies compared to the mesh model. The low-energy AFs in this combination have conventionally been estimated as 0.5 × CF value, assuming that the RBM occupies a fraction of the TB–marrow interface proportional to its CF value (ICRP 2023). While the voxel model aligns well with this assumption, the mesh model yields higher values, suggesting that the RBM occupies a larger portion of the interface than the CF value alone would indicate. As shown in figure 9, the developed mesh models exhibit a nonlinear relationship, particularly at low CF values, due to their anatomically faithful and structurally detailed representation of RBM and YBM compared to the simplified voxel models.

With the TBV as the source region, the voxel model overestimates the values compared to the mesh model at low energies, with differences reaching up to 35%. This overestimation is consistent with the so-called ‘voxel effect’ described in previous predictions (Rajon et al 2002), which found that the stepped voxel geometry exaggerates the TB–marrow interface and thus overestimates electron escape, inflating the values by up to a factor of 2. The difference is smaller when the RBM is the target region, as the larger share of the TB–marrow interface occupied by the RBM in the mesh model, discussed earlier, partially compensates for this overestimation.

For combinations with the RBM and YBM as the source regions and the endosteum as the target region, the two models show excellent agreement across the entire energy range, with differences within 3%. However, noticeable differences arise when the RBM is the target region; at low energies, the voxel model overestimates the values when the RBM is the source region and underestimates them when the YBM is the source region, with maximum differences of up to a factor of 1.8. These trends further reflect the improved representation of RBM and YBM in the mesh model.

**Figure 10.**
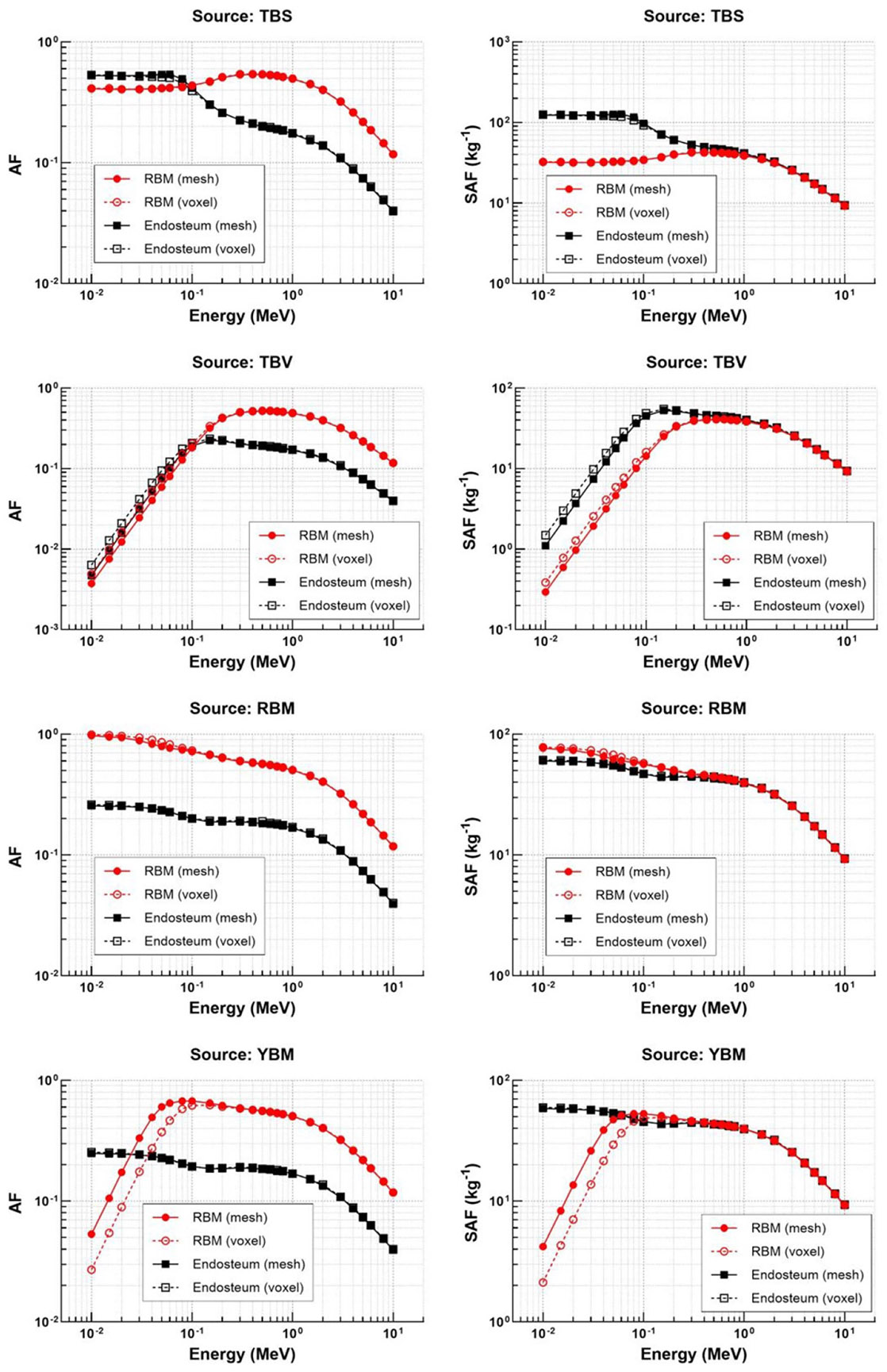
Absorbed fractions (AFs) and specific absorbed fractions (SAFs) calculated using the mesh-based detailed skeletal model (‘mesh’) and its voxelized model (‘voxel’) of the female T6. AFs are shown on the left, SAFs on the right. Source regions are the trabecular bone surface (TBS), trabecular bone volume (TBV), red bone marrow (RBM), and yellow bone marrow (YBM), while target regions are the RBM and endosteum.

Figure 10 presents the values calculated for the female T6 using the mesh and voxel models. As with the male proximal humeri in figure 8, noticeable differences are observed, with the largest reaching up to a factor of 2. Although the overall trends in the differences are similar between the two skeletal sites, the magnitude varies due to differences in the BV/TV and CF values. For example, when the TBS is the source region and the RBM the target region, the two models yield nearly identical results, even at low energies. As shown in figure 9, this is because, at high CF values, the RBM contact fraction in the mesh model closely matches the CF value, resulting in closer agreement between the two models.

#### 3.2.2. Impact of alignment with target skeletal data

**Figure 11.**
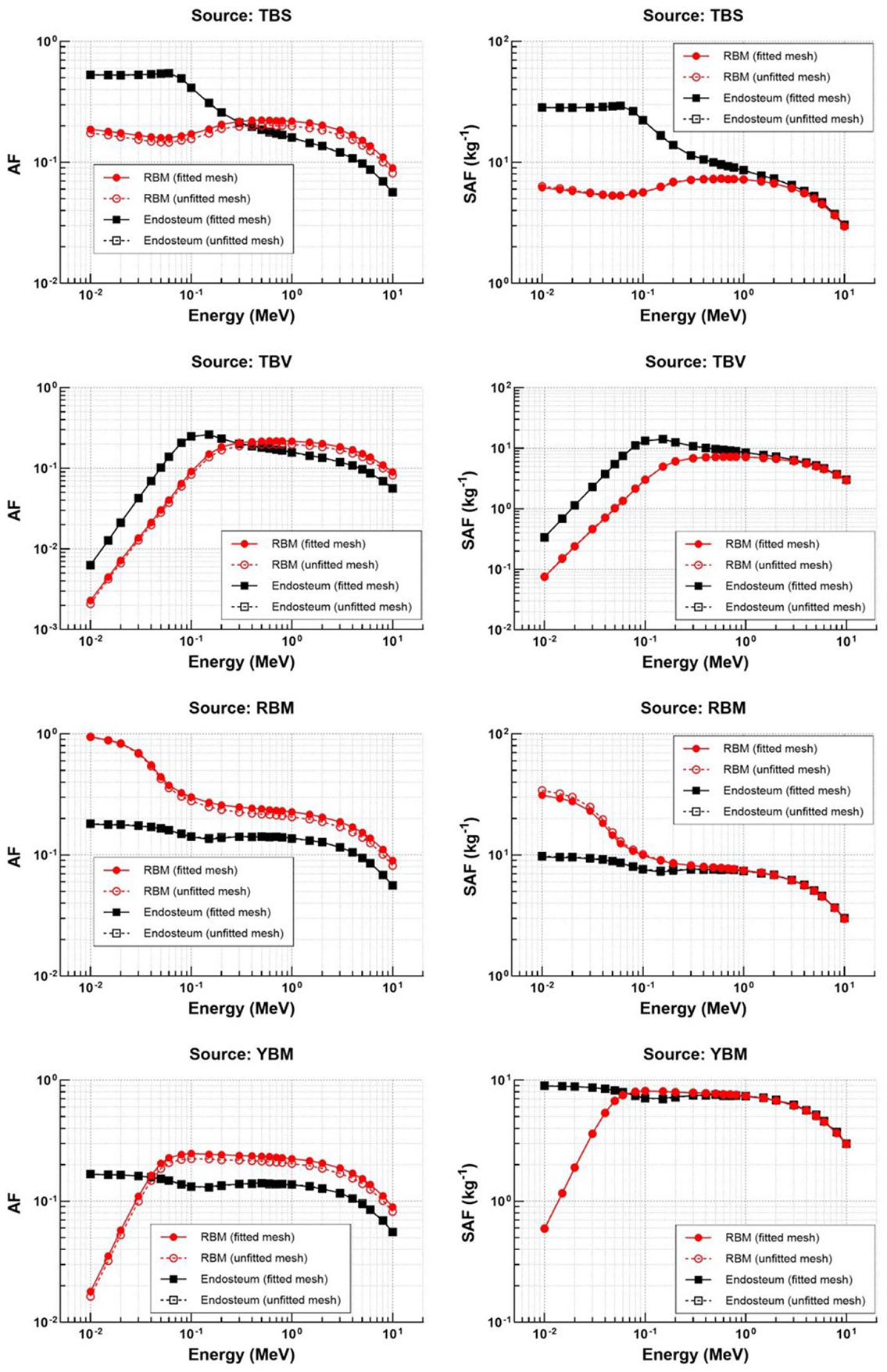
Absorbed fractions (AFs) and specific absorbed fractions (SAFs) calculated using two detailed mesh-based skeletal models of the male proximal humeri: the final model fitted to the target skeletal data (‘fitted mesh’) and another model preserving the original data (‘unfitted’). Source regions are the trabecular bone surface (TBS), trabecular bone volume (TBV), red bone marrow (RBM), and yellow bone marrow (YBM), while target regions are the RBM and endosteum.

Figure 11 presents the values calculated for the male proximal humeri using two mesh models: one fitted to the target skeletal data and the other unfitted. The BV/TV values of the two models are nearly identical (0.087), resulting in excellent agreement across all source region cases when the endosteum is the target region, with maximum differences remaining within ~2%. This outcome is expected, as the definition of the endosteum depends solely on the TB structure. In contrast, the CF values differ, measured at 0.271 for the fitted model and 0.247 for the unfitted model, with the fitted model exhibiting the higher CF value. Consequently, in most combinations where the RBM is the target region, the fitted model yields higher AFs compared to the unfitted model. However, because the RBM target mass is also greater in the fitted model, the resulting SAFs remain largely consistent between the two models. An exception occurs when the RBM is both the source and target region (i.e. self-irradiation), where the low-energy AFs approach unity regardless of the CF value, and the SAFs become approximately inversely proportional to the RBM mass. Therefore, differences in RBM target masses between the fitted and unfitted models lead directly to observable differences in the SAFs for these combinations.

**Figure 12.**
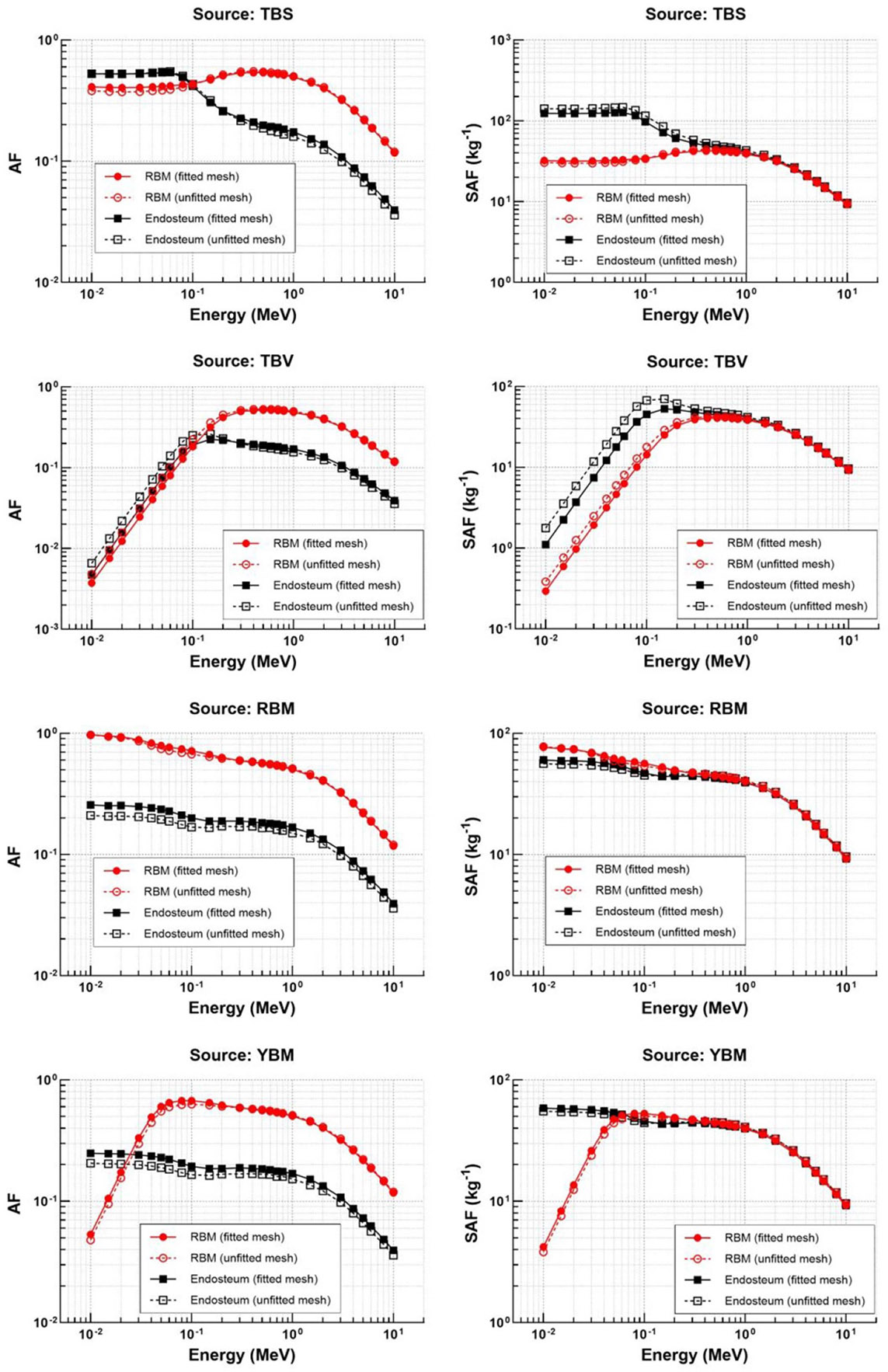
Absorbed fractions (AFs) and specific absorbed fractions (SAFs) calculated using two detailed mesh-based skeletal models of the female T6: the final model fitted to the target skeletal data (‘fitted mesh’) and another model preserving the original data (‘unfitted’). Source regions are the trabecular bone surface (TBS), trabecular bone volume (TBV), red bone marrow (RBM), and yellow bone marrow (YBM), while target regions are the RBM and endosteum.

Figure 12 shows the values calculated for the female T6 using the fitted and unfitted mesh models. Unlike the male proximal humeri case, where the BV/TV values were nearly identical, the fitted model exhibits the substantially higher BV/TV value (0.160) compared to the unfitted model (0.097). The CF value is also higher in the fitted model (0.757) than in the unfitted model (0.707). The higher BV/TV value in the fitted model expands the volume and surface area of the TB, thereby enlarging the endosteum. However, this expansion simultaneously reduces the relative volume of the marrow region, partially offsetting the effect of the higher CF and resulting in similar RBM masses between the two models. As a result, a broader range of differences is observed across all source region cases, with maximum differences reaching up to a factor of 1.6.

For example, when the TBV is the source region, the fitted model yields lower AFs at low energies due to increased self-absorption of electrons within the expanded TB structure. When the endosteum is the target region, the effect of increased endosteal mass further lowers the SAFs, resulting in differences of up to a factor of 1.6. In contrast, when the RBM is the target region, the RBM masses are comparable between the two models, and thus the trends observed in the AF differences are similarly reflected in the SAFs. Another example arises when the RBM is the source region. When the RBM is the target region, the AFs show minimal differences and similar RBM masses lead to similar SAFs. However, when the endosteum is the target region, the fitted model yields higher AFs. This is because the increase in BV/TV value reduces the marrow region while enlarging the endosteum, resulting in a greater fraction of the marrow region being occupied by the endosteum. Nevertheless, the increased endosteal mass in the fitted model partially mitigates this effect, making the differences in SAFs less pronounced than those observed in the AFs.

## 4. Discussion

### 4.1. Improvements, challenges, and implications for skeletal dosimetry

In the present study, we developed 35 male and 38 female mesh-based detailed skeletal models for the ICRP Reference Adults based on the UF *μ*CT images. While several research groups, including UF, have previously developed image-based skeletal models, all prior efforts were limited to the voxel format. These models were inherently constrained by the finite resolution and rigid structure of the voxel format, with limitations such as difficulty in properly representing the detailed and complex morphology of skeletal tissues. To the best of our knowledge, this study represents the first development of detailed skeletal models in the mesh format, recognized as the most advanced approach in computational dosimetry (Kainz et al 2019), significantly advancing the representation of skeletal tissues. It also represents the first attempt to minimize individual anatomical bias, grounded in anatomically reasonable evidence, while ensuring consistency with the ICRP data (ICRP 2002), enabled by the inherent modeling flexibility and deformability of the mesh format.

The dosimetric impact analysis demonstrated that, compared to their voxelized counterparts, the mesh models produced substantial differences in both AFs and SAFs, reaching up to a factor of 2 at low energies (*<*200 keV). Some of these differences were consistent with the previously known ‘voxel effect’ (Rajon et al 2002), whereas others, newly identified in the present study, were attributed to the mesh models’ improved representation of microscopic and complex structures. Additional analysis comparing the fitted mesh model with unfitted counterparts showed notable differences, with the largest reaching up to a factor of 1.6 when the BV/TV values varied substantially. These findings highlight that the developed models, owing to their enhanced geometric accuracy and anatomical fidelity, are expected to serve as a reliable tool for skeletal dosimetry across various exposure scenarios, in combination with the ICRP’s newly released reference phantoms, the MRCPs of ICRP Publication 145 (ICRP 2020b).

However, a key technical challenge remains in applying the developed detailed skeletal models to exposure scenarios requiring whole-body skeletal dosimetry. Their highly refined structures result in an extremely large number of tetrahedra (see section 3.1), leading to substantial file sizes. For example, the file size of the male os coxae, which is the largest among all skeletal sites, is 11.5 GB. While simulation at a single skeletal site is feasible, applying the models across all skeletal sites is impossible at present. Shin et al (2022) applied voxel-based detailed skeletal models (Hough et al 2011, O’Reilly et al 2016) to all skeletal sites of the MRCPs (ICRP 2020b) using the parallel geometry feature of the Geant4 code, marking the first full-skeleton application of such models. Despite their smaller size, the voxel models required 143.4 GB and 112.6 GB of RAM for the male and female, respectively. Given the much larger size of the mesh models developed in the present study, full-skeleton implementation is expected to require at least several, and possibly even tens of terabytes of RAM, exceeding the capacity of most current high-performance computing systems.

To address this challenge, we plan to calculate particle-specific AF datasets for all skeletal sites and use them to produce a new dataset of fluence-to-dose response functions (DRFs). Existing DRFs from ICRP Publication 116 (ICRP 2010), derived using the UF voxel-based male skeletal models (Hough et al 2011) implemented in the UF male phantom (Lee et al 2010), have been applied to both the ICRP male and female reference phantoms, despite being based on the male-only models and not fully aligned with the anatomical data of the ICRP reference phantoms. In contrast, the new DRFs to be generated using the mesh-based models developed in the present study will be anatomically consistent with the MRCPs, account for sex-specific skeletal structures, and reflect improvements over the limitations of voxel models. As a result, users will be able to perform more reliable whole-body skeletal dosimetry without the need to install or manage the detailed skeletal models directly.

### 4.2. Potential application 1: optimization of radiopharmaceutical therapy (RPT)

Although this study focused on developing detailed skeletal models for the ICRP Reference Adults, the skeletal data–based automated modeling techniques established during the development have broader applicability. In particular, they show strong potential for advancing skeletal dosimetry in RPT. In RPT, the RBM is considered a potential absorbed dose-limiting organ at risk, making its accurate dosimetry essential for the safe and effective delivery of treatment. Estimating the RBM absorbed dose involves three key steps (Hindorf et al 2010): (1) quantifying activity uptake in relevant compartments such as blood, bone, or RBM (Sgouros et al 2000); (2) determining patient-specific volume fractions of RBM, YBM, and TB (Pichardo et al 2011); and (3) selecting appropriate S values from parameterized models that incorporate these volume fractions (Geyer et al 2017). Note that the S value is the absorbed dose to the target region per nuclear decay of the radionuclide in the source region, and it can be calculated by combining SAFs with the nuclear decay data.

#### 4.2.1. Imaging-based RBM dosimetry

Quantifying the time-dependent activity uptake is a fundamental prerequisite for RBM dosimetry. For many RPTs, imaging-based dosimetry is essential, as an increasing number of recent studies have demonstrated a clear relationship between absorbed dose and treatment effects in patient populations (Svensson et al 2016, Hagmarker et al 2019, Hemmingsson et al 2023, Blakkisrud et al 2024, Hebert et al 2024, Persson et al 2024). This growing evidence has established imaging-based methodologies as the gold standard for RBM dosimetry. Despite these advances, accurately determining the activity distribution and the active/irradiated volume remains challenging. Many radionuclides used in RPTs, such as the beta-emitters Lu-177 and I-131 and the progeny of the alpha-emitters Ac-225 and Ra-223, feature gamma transitions suitable for imaging with gamma cameras. However, accurately quantifying their activity distribution presents several challenges (Dickson et al 2023): (a) limited spatial resolution, as SPECT/CT systems, typically with centimeter-level resolution, cannot distinguish uptake between YBM, RBM, and TB compartments; and (b) insufficient sensitivity, as low photon counts in SPECT/CT systems lead to poor counting statistics and image reconstruction artifacts, complicating accurate activity quantification.

#### 4.2.2. Determining patient-specific volume fractions

RBM dosimetry is inherently challenging due to the intricate microstructure of TB and the heterogeneous distribution of RBM, which vary significantly across individuals and skeletal sites. Determining patient-specific volume fractions of RBM, YBM, and TB is further complicated by individual variability (e.g. age, sex, and pre-existing conditions) and the limited availability of accurate, non-invasive measurement techniques. In the absence of widely established *in vivo* methods to characterize spongiosa composition, internal dosimetry programs such as IDAC-Dose (Andersson et al 2017) and MIRDcalc (Kesner et al 2023) rely on S values derived from reference volume fractions for absorbed dose calculations. These approaches approximate spongiosa composition using fixed volume fractions, which may not reflect individual patient anatomy. Ongoing research seeks to improve these approximations through advanced imaging techniques, including MRI for assessing marrow cellularity (Pichardo et al 2007, Salas-Ramirez et al 2018) and dual-energy CT (DECT) for evaluating bone composition (Salas-Ramirez et al 2019, 2022), which show promise for enabling more accurate, patient-specific dosimetry.

#### 4.2.3. This study’s contribution

This study supports the development of parameterized, patient-specific mesh-based detailed skeletal models for the third step of RBM dosimetry—selecting appropriate S values tailored to individual patients. When integrated with Monte Carlo transport simulations, these models would enable dynamic calculation of site-specific S values across a wide range of RBM, YBM, and TBV fractions. Unlike conventional approaches that rely on fixed reference values, they would allow integration of patient-specific anatomical data from MRI and DECT. This capability would improve the accuracy of absorbed dosetoxicity correlations, thereby optimizing radiation delivery while reducing toxicity risks. By substantially lowering uncertainties in skeletal dosimetry, the proposed techniques are expected to advance personalized treatment planning in RPT.

### 4.3. Potential application 2: development of mesh-based detailed skeletal models for ICRP reference children

The modeling techniques developed in the present study can also be extended to create mesh-based detailed skeletal models for the ICRP Reference Children (newborn, 1 year, 5 years, 10 years, and 15 years), which can be used in conjunction with the pediatric MRCPs (ICRP 2024). For the pediatric population, the acquisition of high-resolution *μ*CT images is generally impractical due to ethical concerns and the limited availability of pediatric cadaver donations. Accordingly, 3D image-based skeletal models for these age groups are currently unavailable. Thus, pediatric skeletal dosimetry has relied on simplified assumptions, such as homogeneous spongiosa composition, or on the use of pediatric DRFs provided in ICRP Publication 155 (ICRP 2023), which, unlike the adult DRFs, were not directly derived from detailed models but instead obtained through various assumptions and adjustments. By adapting adult *μ*CT images to match pediatric skeletal dimensions such as trabecular number (Tb.N), BV/TV, and CF values obtained from diverse literature sources, the techniques developed in this study could be used to generate anatomically plausible detailed skeletal models for the ICRP Reference Children that reflect non-linear, age-specific skeletal characteristics. These models would then facilitate the derivation of pediatric DRFs based on the detailed skeletal models, thereby enhancing the accuracy of skeletal dosimetry for the pediatric population.

## 5. Conclusion

This study marks the first development of a total of 35 male and 38 female detailed skeletal models in the mesh format, specifically aligned with the ICRP Reference Adults. These models were developed from the UF *μ*CT images through an automated pipeline implemented with in-house C++ and Python programs. To demonstrate the dosimetric impact of the improved model format, AFs and SAFs were compared for representative male and female sites (i.e. the male proximal humeri and female T6), showing up to a twofold improvement compared to voxelized models. The impact of enhanced anatomical fidelity was also investigated, revealing differences of up to a factor of 1.6 between models fitted to the target skeletal data and those unfitted. In future work, we plan to calculate particle-specific AF datasets and produce a new DRF dataset, thereby enabling accurate skeletal dosimetry without requiring users to directly manage the detailed skeletal models. Moreover, the skeletal data–based automated modeling techniques developed in this study are expected to have broader applications, including the optimization of RPT through patient-specific RBM absorbed dose assessments.

## Supplementary Material

2025 Choi PMB - Supplemental Data

Supplementary material for this article is available online

## Figures and Tables

**Table 1.** Details of skeletal sites and dimensions (number of voxels in the *x, y*, and *z* directions) of micro-computed tomography (*μ*CT) images from the University of Florida (UF) (Hough et al 2011, O’Reilly et al 2016). The voxel resolution of all images is isotropically 30 *μ*m.

40 year-old male (Hough et al 2011)		45 year-old female (O’Reilly et al 2016)	
Skeletal site	Dimension	Skeletal site	Dimension
Craniofacial bones		Craniofacial bones	
Frontal/facial	277 × 311 × 51	Frontal/facial	132 × 103 × 17
Parietal	249 × 358 × 49	Parietal	88 × 86 × 17
Occipital	228 × 234 × 81	Occipital	161 × 125 × 12
Mandible	333 × 223 × 59	Mandible	112 × 76 × 56
Scapulae	164 × 228 × 81	Scapulae	90 × 103 × 65
Clavicles	433 × 96 × 106	Clavicles	277 × 177 × 86
Sternum	220 × 177 × 112	Sternum	144 × 142 × 100
Ribs		Ribs	
Upper	388 × 138 × 44	Upper	84 × 39 × 37
Middle	494 × 169 × 53	Lower	96 × 29 × 28
Lower	495 × 108 × 72		
Cervical vertebrae		Cervical vertebrae	
C3	109 × 90 × 75	C3	249 × 134 × 131
C6	358 × 162 × 67	C6	133 × 228 × 142
Thoracic vertebrae		Thoracic vertebrae	
T3	88 × 311 × 158	T1	81 × 63 × 87
T6	338 × 144 × 90	T3	106 × 100 × 91
T11	261 × 177 × 93	T6	100 × 111 × 106
		T9	109 × 109 × 102
		T12	159 × 123 × 137
Lumbar vertebrae		Lumbar vertebrae	
L2	167 × 232 × 112	L1	183 × 148 × 159
L4	189 × 193 × 120	L2	183 × 161 × 148
		L3	217 × 148 × 136
		L4	171 × 183 × 138
		L5	202 × 136 × 158
Sacrum	166 × 181 × 144	Sacrum	140 × 85 × 105
Os coxae	144 × 396 × 76	Os coxae	136 × 103 × 69
Humeri		Humeri	
Proximal	214 × 218 × 94	Proximal	194 × 134 × 127
Distal	141 × 132 × 235	Distal	359 × 104 × 118
Radii		Radii	
Proximal	229 × 176 × 87	Proximal	110 × 120 × 105
Distal	124 × 300 × 118	Distal	223 × 183 × 107
Ulnae		Ulnae	
Proximal	213 × 125 × 164	Proximal	180 × 95 × 120
Distal	201 × 153 × 123	Distal	189 × 152 × 95
Femora		Femora	
Proximal head	151 × 191 × 150	Proximal head	199 × 141 × 157
Proximal neck	178 × 163 × 149	Proximal neck	204 × 42 × 77
Distal	209 × 210 × 99	Distal	158 × 171 × 162
		Patellae	204 × 103 × 81
Tibiae		Tibiae	
Proximal	175 × 182 × 136	Proximal	155 × 146 × 189
Distal	185 × 241 × 98	Distal	189 × 142 × 164
Fibulae		Fibulae	
Proximal	188 × 136 × 172	Proximal	209 × 115 × 115
Distal	155 × 213 × 132	Distal	296 × 153 × 96

**Table 2.** Comparison of expected skeletal tissue masses when micro-computed tomography (*μ*CT) images are directly integrated into spongiosa of mesh-type reference computational phantoms (MRCPs) of ICRP Publication 145 (ICRP 2020b), with values from ICRP Publication 89 (ICRP 2002) for ICRP Reference Adults (i.e. target masses). Target masses include all assigned blood content and miscellaneous tissues for each skeletal tissue, excluding the yellow bone marrow (YBM) mass within the medullary cavity.

Skeletal tissue	Male			Female		
	Expected mass (g)	Target mass (g)	Difference (%)	Expected mass (g)	Target mass (g)	Difference (%)
TB	1149.0	1202.4	4.4	1121.8	879.7	27.5
RBM	1325.4	1449.0	8.5	989.7	1108.8	10.7
YBM	2492.7	2346.3	6.2	1636.2	1652.2	1.0

**Table 3.** Information on collected studies providing bone volume fraction (BV/TV) values from micro-computed tomography (*μ*CT) scans on multiple cadavers without any skeletal adjustments. Detailed regions are specified when BV/TV values are available for those regions.

Reference	Skeletal site (BV/TV mean *±* standard deviation)	N of cadavers^a^
Bevill et al (2006)	Proximal femora	^a^10 (5 males; 5 females)
	Neck^a^ (0.29 ± 0.05)	
	Neck^b^ (0.21 ± 0.05)	^b^10 (5 males; 5 females)
	Greater trochanter^c^ (0.11 ± 0.03)	^c^7 (5 males; 2 females)
	Proximal tibiae^d^ (0.12 ± 0.03)	^d^5 males
	Vertebrae (entire)^e^ (0.10 ± 0.04)	^e^19 (4 males; 15 females)
Boruah et al (2015) ^b^	Craniofacial bones	10 males
	Frontal/facial (0.45 ± 0.21)	
	Parietal (0.52 ± 0.22)	
Chappard et al (2006)	Proximal femora—head (0.23 ± 0.08)	14 (unknown gender)
Eckstein et al (2007) ^c^	Distal radii (male: 0.15 ± 0.05; female: 0.10 ± 0.05)	150 (75 males; 75 females)
	Calcaneus (male: 0.15 ± 0.05; female: 0.14 ± 0.05)	
	Iliac crest (male: 0.07 ± 0.03; female: 0.07 ± 0.04)	
	Lumbar vertebrae—L2 (male: 0.11 ± 0.04; female: 0.10 ± 0.04)	
	Proximal femora	
	Neck (male: 0.18 ± 0.09; female: 0.12 ± 0.07)	
	Trochanter (male: 0.13 ± 0.04; female: 0.11 ± 0.04)	
Gong et al (2006)	Lumbar vertebrae—L4 (0.06 ± 0.02)	6 males
Hulme et al (2007)	Vertebrae (entire) (0.13 ± 0.02)	20 (unknown gender)
Mavroudas and Dominguez (2022)	Middle ribs	^f^40 males ^g^39 males
	50% cutaneous^f^ (0.21 ± 0.06)	
	50% medullary^f^ (0.13 ± 0.06)	
	50% pleural^f^ (0.11 ± 0.04)	
	75% cutaneous^g^ (0.18 ± 0.07)	
	75% medullary^g^ (0.10 ± 0.06)	
	75% pleural^g^ (0.09 ± 0.05)	
Moon et al (2004)	Mandible	10 (7 males; 3 females)
	Alveolar bone (0.44 ± 0.16)	
	Basal bone superior to the mandibular canal (0.20 ± 0.06)	
	Basal bone inferior to the mandibular canal (0.09 ± 0.07)	
Parsa et al (2015)	Mandible (0.32 ± 0.19)	20 (unknown gender)
Perilli et al (2012)	Lumbar vertebrae—L2, L3 (0.06 ± 0.02)	8 (5 males; 3 females)
Rajapakse et al (2010)	Distal tibiae (0.09 ± unknown)	15 (11 males; 4 females)
Schröder et al (2021)	Cervical vertebrae (male: 0.29 ± 0.04; female: 0.29 ± 0.03)	10 (4 males; 6 females)
	Thoracic vertebrae (male: 0.18 ± 0.01; female: 0.19 ± 0.03)	
	Lumbar vertebrae (male: 0.18 ± 0.01; female: 0.16 ± 0.01)	
Schröder et al (2022)	Cervical vertebrae (male: 0.26 ± 0.05; female: 0.21 ± 0.02)	13 (4 male; 9 females)
	Thoracic vertebrae (male: 0.17 ± 0.01; female: 0.17 ± 0.01)	
	Lumbar vertebrae (male: 0.17 ± 0.02; female: 0.15 ± 0.01)	
Ulrich et al (1999)	Calcaneus^h^ (0.12 ± 0.03)	^h^60 (unknown gender)
	Iliac crest^i^ (0.15 ± 0.05)	^i^62 (unknown gender)
	Lumbar vertebrae—L2^j^ (0.08 ± 0.02)	^j^58 (unknown gender)
	Proximal femora—head^k^ (0.21 ± 0.05)	^k^58 (unknown gender)
Van Dessel et al (2013)	Mandible (0.34 ± 0.05)	8 (unknown gender)

a When specified, the number of cadavers used to derive BV/TV is reported as a subset of the total cadavers included in the study.

b The mean and standard deviation of BV/TV were calculated from raw data provided by the authors.

c The mean and standard deviation of BV/TV were extracted from the graph.

**Table 4.** Target bone volume fraction (BV/TV) and cellularity factor (CF) established for mesh-based detailed skeletal models.

Male				Female			
Skeletal site of *μ*CT images	Skeletal site to be applied in this study	Target BV/TV	Target CF	Skeletal site of *μ*CT images	Skeletal site to be applied in this study	Target BV/TV	Target CF
Craniofacial bones	Craniofacial bones			Craniofacial bones	Craniofacial bones		
Frontal/facial	Frontal/facial	0.407	0.422	Frontal/facial	Frontal/facial	0.356	0.413
Parietal	Parietal	0.373	0.422	Parietal	Parietal	0.597	0.413
Occipital	Occipital	0.899	0.422	Occipital	Occipital	0.576	0.413
Mandible	Mandible	0.135	0.422	Mandible	Mandible	0.135	0.413
Scapulae	Scapulae	0.149	0.422	Scapulae	Scapulae	0.040	0.413
Clavicles	Clavicles	0.091	0.366	Clavicles	Clavicles	0.058	0.359
Sternum	Sternum	0.063	0.777	Sternum	Sternum	0.062	0.761
Ribs	Ribs			Ribs	Ribs		
Upper	R1–R4	0.099	0.777	Upper	R1–R6	0.064	0.761
Middle	R5–R8	0.132	0.777	Lower	R7–R12	0.077	0.761
Lower	R9–R12	0.097	0.777				
Cervical vertebrae	Cervical vertebrae			Cervical vertebrae	Cervical vertebrae		
C3	C1–C3	0.215	0.777	C3	C1–C3	0.195	0.761
C6	C4–C7	0.215	0.777	C6	C4–C7	0.195	0.761
Thoracic vertebrae	Thoracic vertebrae			Thoracic vertebrae	Thoracic vertebrae		
T3	T1–T4	0.152	0.777	T1	T1–T2	0.160	0.761
T6	T5–T8	0.152	0.777	T3	T3–T4	0.160	0.761
T11	T9–T12	0.152	0.777	T6	T5–T7	0.160	0.761
				T9	T8–T10	0.160	0.761
				T12	T11–T12	0.160	0.761
Lumbar vertebrae	Lumbar vertebrae			Lumbar vertebrae	Lumbar vertebrae		
L2	L1	0.158	0.777	L1	L1	0.139	0.761
	L2	0.110	0.777	L2	L2	0.079	0.761
	L3	0.079	0.777	L3	L3	0.079	0.761
L4	L4	0.084	0.777	L4	L4	0.084	0.761
	L5	0.158	0.777	L5	L5	0.139	0.761
Sacrum	Sacrum	0.135	0.777	Sacrum	Sacrum	0.113	0.761
Os coxae	Os coxae	0.088	0.533	Os coxae	Os coxae	0.035	0.522
Humeri	Humeri			Humeri	Humeri		
Proximal	Proximal	0.088	0.277	Proximal	Proximal	0.069	0.272
Distal	Distal (hand bones)	0.130	0.000	Distal	Distal (hand bones)	0.119	0.000
Radii	Radii			Radii	Radii		
Proximal	Proximal	0.157	0.000	Proximal	Proximal	0.089	0.000
Distal	Distal	0.098	0.000	Distal	Distal	0.079	0.000
Ulnae	Ulnae			Ulnae	Ulnae		
Proximal	Proximal	0.147	0.000	Proximal	Proximal	0.199	0.000
Distal	Distal	0.135	0.000	Distal	Distal	0.055	0.000
Femora	Femora			Femora	Femora		
Proximal head	Proximal head	0.259	0.277	Proximal head	Proximal head	0.242	0.272
Proximal neck	Proximal remaining	0.099	0.277	Proximal neck	Proximal remaining	0.102	0.272
Distal	Distal (patella, foot bones)	0.125	0.000	Distal	Distal	0.209	0.000
					Foot bones	0.189	0.000
				Patellae	Patellae	0.139	0.000
Tibiae	Tibiae			Tibiae	Tibiae		
Proximal	Proximal	0.090	0.000	Proximal	Proximal	0.118	0.000
Distal	Distal	0.103	0.000	Distal	Distal	0.113	0.000
Fibulae	Fibulae			Fibulae	Fibulae		
Proximal	Proximal	0.067	0.000	Proximal	Proximal	0.090	0.000
Distal	Distal	0.130	0.000	Distal	Distal	0.109	0.000

**Table 5.** PHITS code configurations for Monte Carlo radiation transport simulations used in the calculation of absorbed fractions (AFs).

Item	Detailed skeletal model (mesh)	Detailed skeletal model (voxel)	Mesh-type reference computational phantoms (MRCPs)
Code and version	PHITS version 3.24		
Model implementation	LAT = 3 in [Cell] section (tetrahedral mesh geometry imported)	LAT = 1 in [Cell] section (voxel geometry imported)	LAT = 3 in [Cell] section (tetrahedral mesh geometry imported)
Source generation	s-type = 17 in [Source] section (pre-generated dump file used for all source cases)	s-type = 2 in [Source] section (source generated within voxel geometry) s-type = 17 in [Source] section (pre-generated dump file used for TBS source case)	s-type = 24 in [Source] section (source generated within tetrahedral mesh geometry)
Reflective boundary (infinite geometry)	* in [Surface] section (*-marked surface defined as reflective boundary)	* in [Surface] section (*-marked surface defined as reflective boundary)	Not applied
History numbers	Maxcas = 10 000 and maxbch = 10 in [Parameters] section (100 000 primary electrons transported)		
Physics	negs = 1 in [Parameters] section (EGS5 mode activated)		
Secondary cut value	emin(12,13) = 0.001 and emin(14) = 0.001 in [Parameters] section (1 keV applied for electrons, positrons, and photons)		
Variance reduction	Not applied		
Scoring	Unit = 2 in [T-Deposit] section (energy deposition [MeV/source] in target region stored)		
Post processing	Stored deposited energy divided by primary electron energy to compute absorbed fractions (AFs); AFs further divided by target region mass to compute specific absorbed fractions (SAFs)		

**Table 6.** Comparison of skeletal tissue masses after integrating the developed mesh-based detailed skeletal models into the spongiosa of mesh-type reference computational phantoms (MRCPs) of ICRP Publication 145 (ICRP 2020b), with values from ICRP Publication 89 (ICRP 2002) for ICRP Reference Adults (i.e. target masses).

Skeletal tissue	Male			Female		
	Resulting mass (g)	Target mass (g)	Differences (%)	Resulting mass (g)	Target mass (g)	Differences (%)
TB	1188.2	1202.4	1.2	875.5	879.7	0.5
RBM	1447.5	1449.0	0.1	1111.1	1108.8	0.2
YBM	2355.2	2346.3	0.4	1654.3	1652.2	0.1

**Table 7.** Masses of red bone marrow (RBM) and endosteum for each skeletal site, obtained by integrating the developed mesh-based detailed skeletal models into the spongiosa of mesh-type reference computational phantoms (MRCPs) of ICRP Publication 145 (ICRP 2020b).

Skeletal site (Spongiosa)	Male		Female	
	RBM mass (g)	Endosteum mass (g)	RBM mass (g)	Endosteum mass (g)
Craniofacial bones				
Frontal/facial	20.706	24.734	31.953	42.347
Parietal	39.928	48.071	21.780	42.853
Occipital	4.729	9.033	13.227	20.758
Mandible	16.544	7.387	10.275	3.367
Scapulae	47.639	21.772	35.708	13.519
Clavicles	13.023	3.995	11.253	3.348
Sternum	44.499	9.618	35.412	5.001
Ribs				
R1	11.449	1.792	11.555	2.372
R2	15.226	2.383	14.377	2.952
R3	19.507	3.053	14.613	3.000
R4	26.888	4.209	19.156	3.933
R5	32.629	8.355	20.077	4.122
R6	32.787	8.395	21.297	4.372
R7	35.026	8.969	21.399	3.643
R8	31.718	8.122	19.182	3.265
R9	27.268	6.606	16.171	2.753
R10	27.492	6.660	14.443	2.459
R11	13.137	3.182	9.909	1.687
R12	4.368	1.058	5.804	0.988
Cervical vertebrae				
C1	8.078	3.465	5.295	2.443
C2	7.916	3.396	6.355	2.932
C3	5.580	2.394	5.130	2.367
C4	5.744	2.181	5.442	2.286
C5	5.419	2.057	5.544	2.328
C6	6.234	2.367	6.369	2.675
C7	8.259	3.136	8.081	3.394
Thoracic vertebrae				
T1	15.183	4.800	11.148	4.881
T2	15.562	4.920	11.452	5.014
T3	13.967	4.415	10.472	3.279
T4	13.636	4.311	10.612	3.323
T5	14.540	3.519	11.329	3.824
T6	16.215	3.924	12.600	4.253
T7	17.269	4.179	12.919	4.361
T8	17.831	4.315	14.426	5.225
T9	18.668	5.803	16.011	5.799
T10	21.512	6.687	16.399	5.939
T11	25.080	7.797	17.410	5.141
T12	28.584	8.886	20.860	6.160
Lumbar vertebrae				
L1	36.266	10.491	27.293	7.312
L2	34.550	9.994	29.979	6.780
L3	38.916	11.257	34.306	7.093
L4	40.747	8.092	34.669	8.300
L5	41.039	8.150	34.655	11.663
Sacrum	128.534	36.308	103.339	27.084
Os coxae	277.888	104.414	214.272	43.194
Humeri				
Proximal	30.284	18.679	23.975	14.977
Distal	0.000	9.415	0.000	10.693
Radii				
Proximal	0.000	4.734	0.000	2.265
Distal	0.000	6.700	0.000	4.429
Ulnae				
Proximal	0.000	13.085	0.000	9.861
Distal	0.000	1.336	0.000	0.997
Hand bones	0.000	22.003	0.000	14.756
Femora				
Proximal head	17.692	23.430	19.076	26.633
Proximal remaining	71.741	51.234	34.061	25.304
Distal	0.000	81.244	0.000	46.445
Tibiae				
Proximal	0.000	56.633	0.000	70.829
Distal	0.000	26.913	0.000	30.841
Fibulae				
Proximal	0.000	4.986	0.000	5.727
Distal	0.000	7.798	0.000	6.241
Patella	0.000	6.898	0.000	5.974
Foot bones	0.000	94.065	0.000	71.260
Skeletal site (Medullary cavity)	RBM mass (g)	Endosteum mass (g)	RBM mass (g)	Endosteum mass (g)
Humeri	0	0.901	0	0.714
Radii	0	0.321	0	0.429
Ulnae	0	0.317	0	0.345
Femora	0	1.107	0	1.173
Tibiae	0	0.851	0	1.056
Fibulae	0	0.128	0	0.154
Total	1447.5	891.4	1111.1	704.9

## Data Availability

All data that support the findings of this study are included within the article (and any supplementary information files).
