# Supplementary material for: Mesh-based detailed skeletal models for the ICRP Reference Adults: Part 1. development and dosimetric impact
Source: Phys Med Biol. Author manuscript; Available in PMC 2026 Jul 16. (PMC13373968; doi:10.1088/1361-6560/ae110f)
Supplement: 2025 Choi PMB - Supplemental Data [file NIHMS2190368-supplement-2025_Choi_PMB_-_Supplemental_Data.pdf]

Annex - Detailed Description of the Bone Modeling Algorithm

MESH-BASED DETAILED SKELETAL MODELS FOR THE ICRP REFERENCE ADULTS: PART 1. DEVELOPMENT AND DOSIMETRIC IMPACT

Chansoo Choi[1,4]
Robert J. Dawson[1,4]
Yitian Wang[1]
Bangho Shin[1]
Johannes Tran-Gia[2]
Maikol Salas Ramirez[2]
Anna-Lena Theisen[2]
Michael Lassmann[2]
Wesley E. Bolch[1,3]

[1] J. Crayton Pruitt Family Department of Biomedical Engineering, University of Florida, Gainesville, FL, USA
[2] Department of Nuclear Medicine, University Hospital Würzburg, Würzburg, Germany
[3] Author to whom any correspondence should be addressed.
[4] Authors who contributed equally to this work and are co-first authors of the work.

Acronyms:

TB: trabecular bone

BV/TV: trabecular bone volume fraction

CF: cellularity factor

PM: polygon mesh

TM: tetrahedral mesh

TBV: trabecular bone volume

TBS: trabecular bone surface

RBM: red bone marrow

YBM: yellow bone marrow

The algorithm is organized into several C++ and Python programs which run sequentially, with each program utilizing the output of the previous program. The overall workflow to generate a desired TM model is initially configured and executed using a Bash script.

A.1. Generation of adipocyte block library

A large library of adipocyte models was precomputed and used to procedurally "tile" the marrow cavities of each site-specific bone model. The elements of this library were each constructed within predefined 2 mm length cubic domain for ease of concatenation in three dimensions. This approach also guaranteed that any arbitrary subregion of a TM model (including a subregion within the bone endosteum region, for example) would contain approximately the same cellularity as any other subregion.

The adipocyte size distribution presented in Rozman et al. (1990) is a normal distribution governed by the (unitless) equations

$$\frac{N_V}{\overline{D}} = 47.88 \pm 9.2126$$

and

$$FF = -104.0114 + 2.61293\overline{D} + 0.00838N_V,$$

where N_V is the number of adipocytes per cubic millimeter, $\overline{D}$ is the mean diameter in μm, and FF is the fat fraction (expressed as a percentage) in a healthy bone site. For a fixed fat fraction FF_0, these equations can be solved for the mean and standard deviation of adipocyte diameters as

$$\overline{D} = \frac{FF_0 + 104.0114}{3.01416}$$

and

$$\sigma_D = 0.02561\overline{D}.$$

The relationship between adipocyte size and fat fraction suggested that a library of adipocyte models should be constructed for all possible fat fractions, with varying sizes (and therefore numbers) of adipocytes. However, because of the proportionality between the adipocyte diameter distribution's mean and standard deviation, it was noted that a library of models could instead be constructed for a fixed adipocyte size distribution but different fat fractions, and that isotropic scaling of the appropriately selected model could be performed to achieve any arbitrary adipocyte size distribution and fat fraction combination. Thus, to minimize the size of each model in terms of memory, the size distribution for a high fat fraction (80%) region was chosen for sampling in each adipocyte model.

To generate each adipocyte block, the program used a Poisson disk sampling method to produce a set of points within the cubic domain. For each accepted point (ensuring a minimum distance from its neighbors via a regular spatial grid), a radius was sampled from the normal distribution. An icosahedron was then created at each point with the corresponding radius. All the icosahedra in a

batch were combined into a single mesh object (with facet intersections), which was then passed to CGAL's 3D Alpha Wrapping algorithm to produce a 2-manifold mesh free of intersections or other topological defects. The volume of the resulting mesh was computed (normalized by the domain volume of 8 mm^3) to obtain a volume fraction.

Algorithm 1 Step 0a: Generate Adipocyte Block Candidates

```
procedure GenerateBlocks(domain_size, n_min, n_max, Δn)
    tissue_params ← calculate_params()
    enqueue tasks for n = n_min : Δn : n_max
    spawn worker threads:
    for all batch_size from queue do
        P ← poisson_disk_sampling(tissue_params, batch_size, domain_size)
        M ← ∅
        for all (p, r) in P do
            ico ← generate_icosahedron(p, r)
            M ← M ∪ ico
        end for
        W ← α-wrap(M, α, δ)
        f_vol ← volume(W)/domain_volume
        save_mesh(W, batch_size, f_vol)   makes batch_xxx_vf_yyy.obj
    end for
    join threads
end procedure
```

Several different Poisson exclusion radius criteria were used, expressed in terms of the target adipocyte size distribution's mean. Annex Figure 1 illustrates the behavior of fat (volume) fraction gains with increasing adipocyte (icosahedron) number for different radial exclusion criteria. Data points in this plot correspond to elements of the final adipocyte block library. An additional data series is shown for volume gains with random icosahedral population in space, which was discarded from the methods as it is (1) anatomically unrealistic and (2) unfeasible in terms of computation time and memory demands.

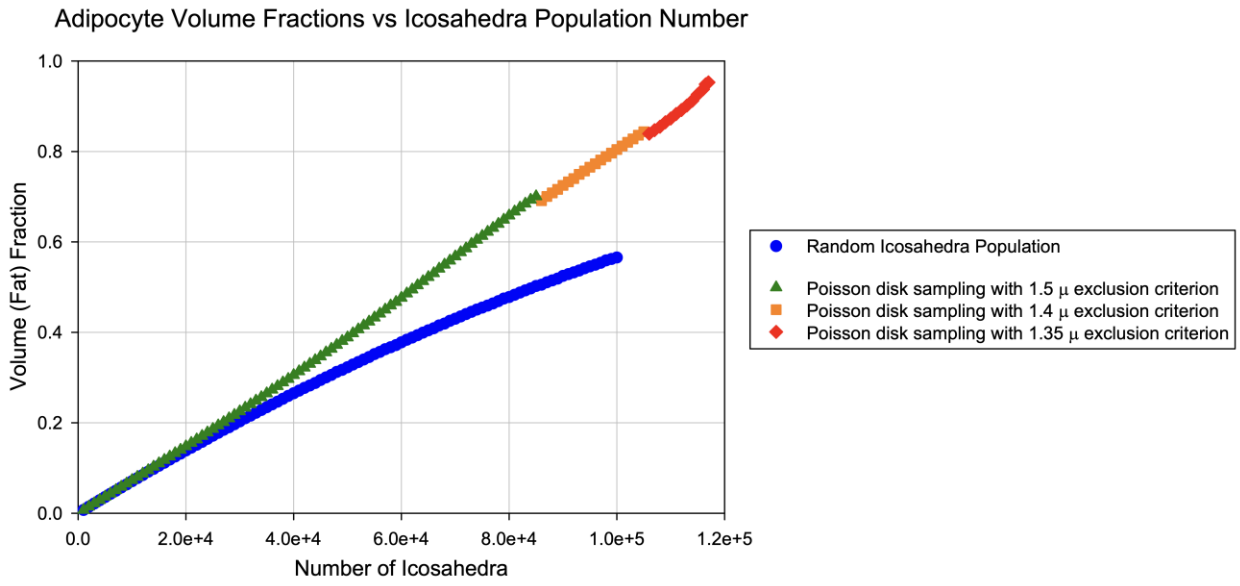

Annex Figure 1. Fat (volume) fraction gains with increasing adipocyte (icosahedron) additions.

A.2. Empirical adjustment model for input tissue parameters

Once the modeling algorithm was finalized, it was observed that the desired values for BV/TV and CF (prescribed in the input shell script) disagreed with the values calculated in the final TM complex by several percent. This difference stemmed from small volume differences between the PM and TM tissue domains introduced during the tetrahedralization process; in particular, for models with a high CF, single PM adipocytes in the marrow region (which were initialized as icosahedra with two additional Catmull-Clark subdivisions) without any local neighbors experienced significant volume changes. These relative volume changes were correlated with the mean size of the adipocytes in the given model as compared to the (constant) *facet_distance* tetrahedral meshing criterion and thus were inconsistent among models with different cellularity factors. Upon further inspection, it also became evident that the input (desired/nominal) BV/TV and CF parameters were correlated with respect to the final model's BV/TV and CF values; for example, for several models (built using the same base TB mesh) with different input values for BV/TV and a shared input value for CF, there was a nonlinear relationship even among the output CF values which were expected to remain constant with changing BV/TV. It therefore became necessary to train a regression model, parametrized by both input BV/TV and CF, which could predict the optimal input values to achieve the desired outputs.

To address this issue, an empirical study was performed in which TM models were generated for the adult male T6 vertebra model with all combinations of input BV/TV and CF values ranging from 0.1 to 0.9, in intervals of 0.1. The BV/TV and CF values computed for these final TM models were then used to derive an initial set of analytical expressions parametrized by the input parameters. This

initial input adjustment model was used to generate the full set of reference bone models, with small perturbations to the nominal BV/TV and CF parameters and concomitant perturbations to the results. These results were then also added to the training data and used to create a new input adjustment model, and this process was iterated until the nominal tissue values closely matched the measured outputs.

The mathematical details of this process are given here. Let the dataset consist of M samples, each with measured quantities

$$\left(T_N^{(m)}, C_N^{(m)}, T_F^{(m)}, C_F^{(m)}\right), \qquad m = 1, \dots, M,$$

where T_N is the nominal BV/TV, C_N is the nominal CF, T_F is the final BV/TV, and C_F is the final CF. For each sample m, the "adjusted" targets are defined as

$$T_A^{(m)} = 2T_N^{(m)} - T_F^{(m)}, \qquad C_A^{(m)} = 2C_N^{(m)} - C_F^{(m)}.$$

These become the regression targets

$$\boldsymbol{y}_T = \left\{T_A^{(m)}\right\}_{m=1}^{M}, \qquad \boldsymbol{y}_C = \left\{C_A^{(m)}\right\}_{m=1}^{M}$$

for input features

$$\boldsymbol{X} = \left\{\left(T_N^{(m)}, C_N^{(m)}\right)\right\}_{m=1}^{M}.$$

Two polynomial functions of degree d (here, $d = 2$) needed to be constructed, $f: \mathbb{R}^2 \to \mathbb{R}$ and $g: \mathbb{R}^2 \to \mathbb{R}$, which give us the mappings $f: (T_N, C_N) \mapsto T_A$ and $g: (T_N, C_N) \mapsto C_A$ of the form

$$f(T_N, C_N) = \sum_{i+j \le d} \theta_{ij}^{(T)} T_N^i C_N^j, \qquad g(T_N, C_N) = \sum_{i+j \le d} \theta_{ij}^{(C)} T_N^i C_N^j$$

with coefficients $\theta_{ij}^{(T)}, \theta_{ij}^{(C)}$ chosen to minimize the ordinary least-squares objectives

$$\min_{\theta^{(T)}} \sum_{m=1}^{M} \left[f\left(T_N^{(m)}, C_N^{(m)}\right) - T_A^{(m)}\right]^2, \qquad \min_{\theta^{(C)}} \sum_{m=1}^{M} \left[g\left(T_N^{(m)}, C_N^{(m)}\right) - C_A^{(m)}\right]^2.$$

This training produced numerical values for $\left\{\theta_{ij}^{(T)}\right\}, \left\{\theta_{ij}^{(C)}\right\}$ and thus the closed-form expressions

$$f(T_N, C_N) = \theta_{00}^{(T)} + \theta_{10}^{(T)} T_N + \theta_{01}^{(T)} C_N + \theta_{20}^{(T)} T_N^2 + \theta_{11}^{(T)} T_N C_N + \theta_{02}^{(T)} C_N^2$$

and

$$g(T_N, C_N) = \theta_{00}^{(C)} + \theta_{10}^{(C)} T_N + \theta_{01}^{(C)} C_N + \theta_{20}^{(C)} T_N^2 + \theta_{11}^{(C)} T_N C_N + \theta_{02}^{(C)} C_N^2.$$

Thus, given any nominal pair of tissue parameters (T_N, C_N), the mapping

$$(T_N, C_N) \mapsto \left(\widehat{T_A}, \widehat{C_A}\right) = \left(f(T_N, C_N), g(T_N, C_N)\right)$$

can be computed, where $\widehat{T_A}, \widehat{C_A}$ are the adjusted inputs predicted to yield the final desired outputs. This process was implemented using the Python modules scikit-learn and Sympy.

Algorithm 2 Step 0b: Train Polynomial Regression Models & Generate Analytical Expressions

procedure TRAINPOLYMODELS(`training_data.csv`, *degree*)
    $D \leftarrow$ read_csv(`training_data.csv`)
    D[Adj_BV/TV] $\leftarrow 2\,D$[Nominal_BV/TV] $- D$[Final_BV/TV]
    D[Adj_CF] $\leftarrow 2\,D$[Nominal_CF] $- D$[Final_CF]
    $\mathbf{X} \leftarrow D$[{Nominal_BV/TV, Nominal_CF}]
    $\mathbf{y_T} \leftarrow D$[Adj_BV/TV], $\mathbf{y_C} \leftarrow D$[Adj_CF]
    $m_T \leftarrow$ train_poly_reg(X, y_T, *degree*)
    $m_C \leftarrow$ train_poly_reg(X, y_C, *degree*)
    compute R_T^2, m_T and R_C^2, m_C
    $e_T \leftarrow$ gen_analytical(m_T, {Nominal_BV/TV, Nominal_CF})
    $e_C \leftarrow$ gen_analytical(m_C, {Nominal_BV/TV, Nominal_CF})
    simplify e_T, e_C
    write expressions to `analytical_expressions.txt`
    return (e_T, e_C)
end procedure

Algorithm 3 Step 0c: Empirical Adjustment of BV/TV and Cellularity

procedure ADJUSTINPUTS(Nominal_BV/TV, Nominal_CF, e_T, e_C)
    Adjusted_BV/TV $\leftarrow e_T$(Nominal_BV/TV, Nominal_CF)
    Adjusted_CF $\leftarrow e_C$(Nominal_BV/TV, Nominal_CF)
    return (Adjusted_BV/TV, Adjusted_CF)
end procedure

A.3. Trabecular bone volume fraction (BV/TV) modulation

The algorithm is initialized with a “cropping factor,” between 0 and 1, which truncates the input trabecular PM model near its boundaries. The input trabecular PM model’s bounding box was constructed as a simple mesh, uniformly scaled along all three dimensions about its centroid by the cropping factor, and then the Boolean intersection of this mesh box and the trabecular mesh is computed. By default, and for all bone site-specific models presented in this study, this parameter was set to 0.9, meaning the region of interest (ROI) that is to be used to construct the TM model excludes 5% of the input trabecular surface on all six sides of the ROI. This is done to ensure flat boundaries in the TM model which are later necessary for accurate internal reflection conditions in radiation transport simulations using PHITS.

A.3.1. Increase BV/TV

This program is run if the current BV/TV is less than the target BV/TV. The algorithm reads in the initial trabecular mesh and constructs a scaled version of its bounding box according to the cropping factor. It then applies CGAL’s 3D Alpha Wrapping with increasing offset parameters, in parallel, until

the wrapped geometry attains at least the target BV/TV. Once this condition is met, the optimized mesh is saved.

Algorithm 4 Step 1a: Increase BV/TV via 3D Alpha Wrapping Optimization

procedure IncreaseBV/TV(Target_BV/TV, *crop*, α)
  $\mathcal{M} \leftarrow$ read_mesh(`Trabeculae.obj`)
  $\mathcal{B} \leftarrow$ create_scaled_bbox($\mathcal{M}$, *crop*)
  $c \leftarrow$ compute_BV/TV($\mathcal{M}$, $\mathcal{B}$) ▷ BV/TV := volume($\mathcal{M} \cap \mathcal{B}$)/volume($\mathcal{B}$)
  if $c \geq$ Target_BV/TV **then**
    return $\mathcal{M}$
  end if
  $\delta \leftarrow \delta_{\min}$ ▷ Surface offset parameter for 3D Alpha Wrapping algorithm
  while $\delta \leq \delta_{\max}$ **do**
    parallel for $i = 0, \ldots, T-1$ **do**
      $\delta_i \leftarrow \delta + i\,\Delta\delta$
      $\mathcal{M}_i \leftarrow \alpha$-wrap($\mathcal{M}, \alpha, \delta_i$)
      $c_i \leftarrow$ compute_BV/TV($\mathcal{M}_i, \mathcal{B}$)
    end parallel
    if any $c_i \geq$ Target_BV/TV **then**
      choose $\mathcal{M}^*$ with min{$|c_i -$ Target_BV/TV$|$}
      write $\mathcal{M}^*$ to `Trabeculae.obj`
      return
    end if
    $\delta \leftarrow \delta + T\,\Delta\delta$
  end while
end procedure

A.3.2. Reduce BV/TV

This program is run if the current BV/TV is greater than the target BV/TV. The algorithm samples a large cloud of interior points from the tetrahedral mesh via barycentric sampling. In this approach, the volume of an individual tetrahedron with vertices $\boldsymbol{t}_0, \boldsymbol{t}_1, \boldsymbol{t}_2, \boldsymbol{t}_3 \in \mathbb{R}^3$, is calculated as

$$V = \frac{1}{6}\left|\det\begin{pmatrix} x_{\boldsymbol{t}_0} & y_{\boldsymbol{t}_0} & z_{\boldsymbol{t}_0} & 1 \\ x_{\boldsymbol{t}_1} & y_{\boldsymbol{t}_1} & z_{\boldsymbol{t}_1} & 1 \\ x_{\boldsymbol{t}_2} & y_{\boldsymbol{t}_2} & z_{\boldsymbol{t}_2} & 1 \\ x_{\boldsymbol{t}_3} & y_{\boldsymbol{t}_3} & z_{\boldsymbol{t}_3} & 1 \end{pmatrix}\right|.$$

To generate a point, a random tetrahedron is selected from all tetrahedra in the domain of interest with a probability proportional to its volume, normalized to the cumulative volume of all tetrahedra. A random Cartesian point is then generated using the well-studied barycentric method as applied to a 3-simplex, and this process is repeated 10^8 times so that a low-noise PM surface can be generated later.

The algorithm then iteratively filters this point set by distance thresholds from the original surface and applies 3D Alpha Wrapping to each filtered subset, again seeking the BV/TV that most closely matches the target. If any candidate point cloud achieves a BV/TV that meets or falls below the target,

that mesh is saved and the program exits; if not, the process is repeated with a more aggressive (higher) distance threshold range, while keeping track of the best result. The entire process is parallelized to accelerate point filtering and candidate evaluation.

Algorithm 5 Step 1b: Reduce BV/TV via Point-Cloud Sampling

procedure ReduceBV/TV(Target_BV/TV, *crop*, α, δ, $N_{\max}$)
  $\mathcal{M} \leftarrow$ read_mesh(`Trabeculae.obj`)
  $\mathcal{B} \leftarrow$ create_scaled_bbox($\mathcal{M}$, *crop*)
  $c \leftarrow$ compute_BV/TV($\mathcal{M}$, $\mathcal{B}$)
  if $c \leq$ Target_BV/TV **then**
    return $\mathcal{M}$
  end if
  $\mathcal{P} \leftarrow$ sample_points_in_tets(`Trabeculae.node`, `.ele`, $N_{\max}$) ▷ 3-simplex barycentric coordinate system
  $t \leftarrow t_{\min}$ ▷ Threshold rejection distance
  loop
    $\{t_i\} \leftarrow \{\, t + j\,\Delta t \mid j = 0, \ldots, K-1\}$
    parallel for each t_i **do**
      $\mathcal{P}_i \leftarrow \{p \in \mathcal{P} \mid \mathrm{dist}(p, \mathcal{M}) \geq t_i\}$
      $\mathcal{M}_i \leftarrow \alpha$-wrap($\mathcal{P}_i$, α, δ)
      $c_i \leftarrow$ compute_BV/TV($\mathcal{M}_i$, $\mathcal{B}$)
    end parallel
    choose $\mathcal{M}^*$ minimizing $|c_i -$ Target_BV/TV$|$
    if compute_BV/TV($\mathcal{M}^*$, $\mathcal{B}$) $\leq$ Target_BV/TV **then**
      write $\mathcal{M}^*$ to `Trabeculae.obj`
      return
    end if
    $t \leftarrow t + K\,\Delta t$
  end loop
end procedure

A.4. Generation of endosteal surface and auxiliary polygon surfaces

Taking the final trabecular mesh as input, the algorithm spawns two parallel processes to produce the trabecular shell and the inner endosteum shell by 3D Alpha Wrapping with prescribed offsets. Once those complete, it wraps the inner shell to create the bulk endosteum mesh and then wraps that mesh again to form the outer endosteum shell. These shells meshes are necessary to create non-ambiguous interface definitions in the next step of the workflow and are set to lie at 1-μm offsets with respect to the trabecular and endosteum PM layers.

Algorithm 6 Step 2: Generate Endosteum and Support Shells

procedure MakeShells(α, δ)
  $\mathcal{M} \leftarrow$ `Trabeculae.obj`
  $\delta_{\text{inner}} \leftarrow 50\,\mu\text{m} - \delta$ ▷ All shells have δ set to $1\,\mu\text{m}$
  spawn **thread** T_1: "Trabeculae_shell.obj" $\leftarrow$ generate_wrapper ($\mathcal{M}$, α, δ)
  spawn **thread** T_2: "Endosteum_shell_inner.obj" $\leftarrow$ generate_wrapper ($\mathcal{M}$, α, δ_{inner})
  join T_1, T_2
  "Endosteum.obj" $\leftarrow$ generate_wrapper ("Endosteum_shell_inner.obj", α, δ)
  "Endosteum_shell_outer.obj" $\leftarrow$ generate_wrapper ("Endosteum.obj", α, δ)
end procedure

A.5. Selection of adipocyte block model

This algorithm selects the best candidate "adipocyte block" based on how closely its final volume fraction, after a series of geometric transformations and Boolean operations, matches the target fat fraction. In this step, a variable called "simple" is introduced and serves as a mode indicator; 0 indicates normal adipocyte processing, while 1 and 2 indicate a CF of 0 or 1. In "simple" (nonzero) modes, the program produces dummy output files and exits; otherwise, it proceeds with full processing.

Exact point property maps are instantiated for use in constructive solid geometry (CSG) computations, which circumvent common geometric issues when performing mesh Boolean operations that arise due to floating point arithmetic errors. The program reads the trabecular mesh and computes a scaling ratio based on the target fat fraction as described previously. It then scans the directory of candidate adipocyte block meshes, extracting a volume fraction from each file's name and selecting the two closest candidates based on the absolute difference from the target value. For each candidate, the block mesh is scaled by the computed ratio and then positioned into a regular 3D grid covering the bounding box. These individual blocks are merged in parallel using Boolean union operations to create a "stacked" mesh. This stacked mesh is intersected with the bounding box and then subtracted by the trabeculae mesh to yield a final mesh whose volume fraction is computed. The candidate whose final volume fraction is closest to the target is saved as the best match, and the program writes detailed parameters and results to a text file.

Algorithm 7 Step 3: Select Optimal Adipocyte Block

procedure SELECTBLOCK(f_{target}, *simple*, $N_{\text{candidates}}$)
if *simple* $\neq 0$ **then**
write dummy `best_block.obj` result file
return
end if
$\mathcal{T} \leftarrow$ read_mesh(`Trabeculae.obj`)
$\mathcal{B} \leftarrow$ read_mesh(`bbox.obj`)
$V_{\mathcal{B}} \leftarrow \text{vol}(\mathcal{B})$
$r \leftarrow$ calculate_scale_ratio(f_{target}) ▷ Scale by the ratio of adipocyte size distribution means (target/initial)
$c \leftarrow \text{centroid}(\mathcal{B})$
$A \leftarrow \emptyset$
for all filename in `adipocyte_block_library` **do**
$f_{\text{initial}} \leftarrow$ extract_volume_fraction(*filename*)
$A \leftarrow$ (*filename*, f_{initial}, r)
end for
Sort A by ascending $|f_{\text{initial}} - f_{\text{target}}|$
$C \leftarrow \{a_i \in A \mid i = 0, \ldots, N_{\text{candidates}}\}$ ▷ Pick top $N_{\text{candidates}}$ blocks from library in terms of volume fraction
$min\_error \leftarrow +\infty$
for all (*filename*, f_{initial}, r) $\in C$ **do**
$\mathcal{M} \leftarrow$ read_mesh(*filename*) ▷ Adipocyte size distribution is still incorrect
$\mathbf{T}_{\text{scale}} \leftarrow \text{translate}(-c) \circ \text{scale}(r) \circ \text{translate}(c)$
$\mathcal{M}_{\text{scaled}} \leftarrow \text{transform}(\mathbf{T}_{\text{scale}}, \mathcal{M})$ ▷ Isotropically scaled block now has correct size distribution
$l \leftarrow$ side length of cubic adipocyte blocks (2 mm)
$s \leftarrow l\, r$
$\mathcal{V}(\mathcal{B}) \leftarrow \{(x_i, y_i, z_i)\}_{i=1}^{N}$ are the vertices of the global bounding box
$\mathcal{B}_{\min} \leftarrow (\min_i x_i, \min_i y_i, \min_i z_i), \quad \mathcal{B}_{\max} \leftarrow (\max_i x_i, \max_i y_i, \max_i z_i)$
$\Delta\mathcal{B} = (\Delta\mathcal{B}_x, \Delta\mathcal{B}_y, \Delta\mathcal{B}_z) \leftarrow \mathcal{B}_{\max} - \mathcal{B}_{\min}$
$(n_x, n_y, n_z) \leftarrow \lceil (\Delta\mathcal{B}_x, \Delta\mathcal{B}_y, \Delta\mathcal{B}_z)/s \rceil$ ▷ Integer dimensions of block grid
$$(o_x, o_y, o_z) \leftarrow \left(x_{\min} + \frac{\Delta\mathcal{B}_x - n_x s}{2}, y_{\min} + \frac{\Delta\mathcal{B}_y - n_y s}{2}, z_{\min} + \frac{\Delta\mathcal{B}_z - n_z s}{2} \right)$$
$S \leftarrow \emptyset$
for $i = 0, \ldots, n_x - 1$ **do** ▷ Precompute offset vector $\mathbf{o} = (o_x, o_y, o_z)$ so grid is centered on $\mathcal{B}$
for $j = 0, \ldots, n_y - 1$ **do**
for $k = 0, \ldots, n_z - 1$ **do**
$\mathbf{\Delta} = \mathcal{B}_{\min} + (o_x, o_y, o_z) + s\,(i, j, k)$
$\mathbf{T}_{\text{trans}} \leftarrow \text{translate}(\mathbf{\Delta})$
$\mathcal{M}_{i,j,k} \leftarrow \text{transform}(\mathbf{T}_{\text{trans}}, \mathcal{M}_{\text{scaled}})$
$S \leftarrow S \cup \{\mathcal{M}_{i,j,k}\}$
end for
end for
end for
$\mathcal{M}_{\text{stacked}} \leftarrow$ parallel reduce $\bigcup_{\mathcal{X} \in S} \mathcal{X}$ using repeated corefine_and_compute_union until one mesh remains
$\mathcal{M}_{\text{cropped}} \leftarrow \mathcal{M}_{\text{stacked}} \cap \mathcal{B}$ via corefine_and_compute_intersection
$\mathcal{M}_{\text{final}} \leftarrow \mathcal{M}_{\text{cropped}} \setminus \mathcal{T}$ via corefine_and_compute_difference
$$f_{\text{final}} \leftarrow \frac{\text{vol}(\mathcal{M}_{\text{final}})}{\text{vol}(\mathcal{B}) - \text{vol}(\mathcal{T} \cap \mathcal{B})}$$
if $|f_{\text{final}} - f_{\text{target}}| \leq min\_error$ **then**
Save $\mathcal{M}_{\text{final}}$ as `best_block.obj` and record parameters
$min\_error \leftarrow |f_{\text{final}} - f_{\text{target}}|$
end if
end for
end procedure

A.6. Construction of mesh domains and tetrahedralization

This program generates the final TM model through a series of Boolean operations and geometric transformations, again with the "simple" flag as an additional prescription. In the full workflow, the program first reads the input meshes (adipocyte block, trabeculae, trabeculae and endosteum shells, bounding box, and endosteum). It then computes Boolean intersections (to crop the endosteum) and reads the adipocyte block scaling ratio calculated in the previous step. Using the scale ratio, the program calculates a grid over the bounding box and "stacks" copies of the adipocyte block mesh by translating each copy to its corresponding grid cell. These blocks are then merged by repeatedly performing parallel Boolean unions until a single stacked mesh is obtained. Subsequent Boolean difference and intersection operations are used to subtract the trabeculae and to partition the adipocyte mesh into "shallow" and "deep" regions. These subregions are defined symbolically as

$$\underbrace{(Adips_{stacked} - Endosteum_{outer\ shell})}_{Deep\ YBM} \cup \left[\underbrace{(Adips_{stacked} \cap Endosteum_{inner\ shell}) - Trabec_{shell}}_{Shallow\ YBM}\right].$$

Final surface meshes (for trabeculae, endosteum, and adipocytes) are written out, and property maps are cleared to free memory. A bounding box for these meshes is created to define active marrow (RBM), which serves as a "fill" region outside of all other PM-defined regions The final meshes are converted to CGAL polyhedra and collected into a patches vector. The program then creates a mesh domain from these patches using predefined incident subdomain labels and tetrahedralizes the domain to generate a TM complex, which is written to a VTU file.

Algorithm 8 Step 4: Domain Construction & Tetrahedralization

procedure BUILDTETRAHEDRALPHANTOM(*simple*)
  if $simple \neq 0$ **then**
    write dummy `out.vtu`
    return
  end if
  $\mathcal{B} \leftarrow$ read_mesh(`bbox.obj`) ▷ Load all previously generated meshes
  $\mathcal{M}_{\text{adip\_block}} \leftarrow$ read_mesh(`best_block.obj`)
  $\mathcal{M}_{\text{trabec}} \leftarrow$ read_mesh(`Trabeculae.obj`)
  $\mathcal{M}_{\text{endo}} \leftarrow$ read_mesh(`Endosteum.obj`)
  $\mathcal{M}_{\text{trabec\_shell}} \leftarrow$ read_mesh(`Trabeculae_shell.obj`)
  $\mathcal{M}_{\text{endo\_inner}} \leftarrow$ read_mesh(`Endosteum_shell_inner.obj`)
  $\mathcal{M}_{\text{endo\_outer}} \leftarrow$ read_mesh(`Endosteum_shell_outer.obj`)
  Repeat adipocyte block concatenation as in previous step, this time using the optimal $\mathcal{M}_{\text{adip\_block}}$:
  $\mathcal{M}_{\text{adips}} \leftarrow \bigcup_{i,j,k}\{\mathcal{M}_{i,j,k} \mid 0 \leq i \leq n_x - 1,\ 0 \leq j \leq n_y - 1,\ 0 \leq k \leq n_z - 1\}$

  for all domain indices d in `incident_subdomains` **do** ▷ Refine meshes at VOI boundaries
    $\delta_d \leftarrow$ offset to prevent numerical errors near boundaries of $\mathcal{B}$
    $\mathcal{B}_d \leftarrow$ offset_mesh($\mathcal{B}$, δ_d)
    Select input mesh $\mathcal{M}_d$ ($\mathcal{M}_{\text{trabec}}$, $\mathcal{M}_{\text{endo}}$, or $\mathcal{M}_{\text{adips}}$)
    $\mathcal{M}_d \leftarrow$ corefine_and_compute_intersection($\mathcal{M}_d$, $\mathcal{B}_d$)
  end for
  $\mathcal{M}_{\text{adips\_deep}} \leftarrow \mathcal{M}_{\text{adips}}$ ▷ Partition adipocytes into the two disjoint spatial domains
  $\mathcal{M}_{\text{adips\_shallow}} \leftarrow \mathcal{M}_{\text{adips}}$
  $\mathcal{M}_{\text{adips\_deep}} \leftarrow$ corefine_and_compute_difference($\mathcal{M}_{\text{adips}}, \mathcal{M}_{\text{endo\_outer}}$)
  $\mathcal{M}_{\text{adips\_shallow}} \leftarrow$ corefine_and_compute_intersection($\mathcal{M}_{\text{adips}}, \mathcal{M}_{\text{endo\_inner}}$)
  $\mathcal{M}_{\text{adips\_shallow}} \leftarrow$ corefine_and_compute_difference($\mathcal{M}_{\text{adips\_shallow}}, \mathcal{M}_{\text{trabec\_shell}}$)
  $\{\mathcal{M}_d\} \leftarrow \{\mathcal{M}_{\text{trabec}}, \mathcal{M}_{\text{endo}}, \mathcal{M}_{\text{adips\_deep}}, \mathcal{M}_{\text{adips\_shallow}}, \mathcal{B}\}$ ▷ Unoccupied space in $\mathcal{B}$ is labeled as RBM
  $C3t3 \leftarrow$ make_mesh_3($\{\mathcal{M}_d\}$, mesh_criteria) ▷ Tetrahedralize the assembled polyhedral complex
  write $C3t3$ to `out.vtu`
end procedure

A.7. Tetrahedral mesh format conversion

The initial tetrahedral mesh complex for each site-specific skeletal model is generated and written as an unstructured grid in VTU format. While this format contains all node and tetrahedron connectivity information necessary to fully define the TM model, it is not an acceptable geometry input format for Monte Carlo radiation transport codes such as PHITS. Therefore, a program was developed to convert the data into NODE/ELE format, the native file format for TetGen.

The program first loads the entire VTU file into a string and locates the <Piece> element to extract the number of points and cells. It then parses the points from the <Points><DataArray> section, storing them as 3D coordinates, and extracts cell data –connectivity, offsets, and cell types – from the <Cells> section. It also reads per-cell domain labels from the <CellData> section.

Using the connectivity, offsets, and types (only tetrahedra, type 10 in the VTU format, are processed), the program constructs a list of tetrahedral elements; each element is stored with its four vertex indices and an associated domain label. It then computes the volume of each tetrahedron and accumulates the volume per domain. The program writes the point coordinates to a NODE file and

the tetrahedral connectivity with domain labels to an ELE file. Finally, it calculates overall mesh volume, BV/TV, and CF, and outputs these values to a text file.

Algorithm 9 Step 5: Convert VTU to NODE/ELE

---

procedure CONVERTVTU(`out.vtu`)
    parse `NumberOfPoints`, `NumberOfCells` from `<Piece>` tag
    $\mathcal{P} \leftarrow$ parse_Points(`out.vtu`)
    $\mathcal{E} \leftarrow$ parse_Cells(`out.vtu`)
    write `converted.node` with $\mathcal{V}$ (vertex positions)
    write `converted.ele` with $\mathcal{E}$ (connectivity, domain indices)
end procedure

A.8. Creation of dump sources for Monte Carlo radiation transport simulations

Due to the microscopic geometric features in the phantoms, the source point source sampling method used in PHITS failed and prevented the simulation from compiling for tetrahedral volume sources. Furthermore, PHITS does not (yet) provide native support for surface sampling on TM geometry, which is necessary to create the TBS sources for each model. Therefore, following TM model generation, the modeling workflow also generates dump files to serve as source terms in PHITS simulations.

The NODE and ELE files are loaded to recover the nodal coordinates and tetrahedral connectivity. A surface sampling routine enumerates all triangular faces, weights them by area, and randomly selects points on those faces to produce a set of uniformly distributed surface points. A volume sampling routine then samples barycentric points inside the tetrahedra for TB, RBM, and YBM domains – conditionally skipping marrow types according to the “simple” flag – and writes out the corresponding dump files for use as Monte Carlo source definitions.

Algorithm 10 Step 6: Create Surface & Volume Source Files

---

procedure MAKESOURCEDUMPS(N, *simple*)
    $(\mathcal{P}, \mathcal{E}) \leftarrow$ read_mesh(`converted.node`, `.ele`)
    // Surface sampling (always)
    $\mathcal{F} \leftarrow$ extract_triangles($\mathcal{P}, \mathcal{E}$)
    sample N barycentric points on $\mathcal{F}$ weighted by area; write `TBS.dump` ▷ Trabecular surface
    if *simple* $\neq 2$ **then**
        sample N barycentric points in domains $\{2, 5\}$; write `RBM.dump` ▷ Red marrow volume
    end if
    if *simple* $\neq 1$ **then**
        sample N barycentric points in domains $\{3, 4\}$; write `YBM.dump` ▷ Yellow marrow volume
    end if
end procedure
